# Supplementary material for: Scalable semitransparent organic solar cells with robust film thickness tolerance for building-integrated photovoltaics
Source: Nat Commun. 2026 Feb 18;17:2916. doi: 10.1038/s41467-026-69537-3 (PMC13031838; doi:10.1038/s41467-026-69537-3)
Supplement: Supplementary file 1 — Supplementary Information [file 41467_2026_69537_MOESM1_ESM.pdf]

Supplementary Information

Scalable Semitransparent Organic Solar Cells with Robust Film Thickness Tolerance for Building-Integrated Photovoltaics

Tong Wang, Jin Fang, Hao Zhang, Chenyang Tian, Yuhan Wang, Zhen Fu, Wenjun Zou, Dan Deng, Xiaotao Hao, Chang He, Jianqi Zhang*, Zhixiang Wei*

This file includes:

Supplementary Figure 1 | J-V curves of 1 cm^2 opaque and semitransparent PM6:Qx-p-4Cl devices processed by spin coating and slot-die coating.

Supplementary Figure 2 | J-V curves of 1 cm^2 opaque PM6:Y6 devices processed by spin coating and slot-die coating.

Supplementary Figure 3 | J-V curves of 1 cm^2 opaque PM6: L8-BO devices processed by spin coating and slot-die coating.

Supplementary Figure 4 | J-V curves of 1 cm^2 opaque PM6:Qx-1 devices processed by spin coating and slot-die coating.

Supplementary Figure 5 | The PCE distribution at different D:A ratios of spin- and slot-die-coated 1 cm^2 opaque devices based on different systems.

Supplementary Figure 6 | EQE spectra of 1 cm^2 opaque PM6:Qx-p-4Cl devices processed by spin coating and slot-die coating.

Supplementary Figure 7 | EQE spectra of 1 cm^2 opaque PM6:Y6 devices processed by spin coating and slot-die coating.

Supplementary Figure 8 | EQE spectra of 1 cm^2 opaque PM6: L8-BO devices processed by spin coating and slot-die coating.

Supplementary Figure 9 | EQE spectra of 1 cm^2 opaque PM6:Qx-1 devices processed by spin coating and slot-die coating.

Supplementary Figure 10 | EQE and transmittance spectra of 1 cm^2 slot-die-coated semitransparent PM6:Qx-p-4Cl devices.

Supplementary Figure 11 | The transmittance spectra of PM6:Qx-p-4Cl 1 cm^2 slot-die-coated semitransparent devices without MoO_3 ARC.

Supplementary Figure 12 | The EQE, reflection and transmittance spectra of PM6:Qx-p-4Cl 1 cm^2 slot-die-coated semitransparent devices with ARC.

Supplementary Figure 13 | The transmittance spectra of PM6:Qx-p-4Cl (1:1.5) spin-coated active layer and PM6:Qx-p-4Cl (1:3) slot-die-coated active layer with different active layer thickness.

Supplementary Figure 14 | Photographs of a 1 cm^2 slot-die-coated semitransparent device.

Supplementary Figure 15 | The PCE and LUE of ST-OSCs with different active layer thicknesses, and mobility calculation.

Supplementary Figure 16 | 2D GIWAXS patterns.

Supplementary Figure 17 | 1D cut profiles from 2D GIWAXS.

Supplementary Figure 18 | The AFM images of spin- and slot-die-coated PM6:Qx-p-4-Cl blends with different D:A ratios.

Supplementary Figure 19 | The fibril width of slot-die-coated PM6:Qx-p-4Cl blends with different D:A ratios measured from AFM images.

Supplementary Figure 20 | The fibril width of slot-die-coated PM6: Y6 blends with different D:A ratios measured from AFM images.

Supplementary Figure 21 | The fibril width of slot-die-coated PM6:L8-BO blends with different D:A ratios measured from AFM images.

Supplementary Figure 22 | The fibril width of slot-die-coated PM6:Qx-1 blends with different D:A ratios measured from AFM images.

Supplementary Figure 23 | TA spectra for spin-coated PM6:Qx-p-4Cl blends with D:A ratios of 1:0.5 and 1:3.

Supplementary Figure 24 | TA spectra for slot-die-coated PM6:Qx-p-4Cl blends with D:A ratios of 1:1 and 1:5.

Supplementary Figure 25 | The summary of exciton lifetimes.

Supplementary Figure 26 | Fluence dependent TRPL spectra.

Supplementary Figure 27 | The PL and TRPL spectra of Qx-p-4Cl system.

Supplementary Figure 28 | The PL spectra of PM6:Y6, PM6:L8-BO and PM6:Qx-1 with different D:A ratios.

Supplementary Figure 29 | TRPL spectra of PM6:Y6, PM6:L8-BO and PM6:Qx-1 with different D:A ratios.

Supplementary Figure 30 | The transmittance spectra of ST-modules.

Supplementary Figure 31 | CIE-1931 chromaticity diagrams of PM6:Qx-p-4Cl semitransparent devices and modules fabricated in this work.

Supplementary Figure 32 | The detailed size of 100 cm^2 module.

Supplementary Figure 33 | The summary of LUE of reported ST-modules.

Supplementary Figure 34 | The summary of CTM of PCE of reported large-area modules.

Supplementary Figure 35 | The block diagrams of applications of power generation.

Supplementary Figure 36 | Photographs of applications of power generation.

Supplementary Figure 37 | Photograph of ambient temperature.

Supplementary Figure 38 | Operational stability testing.

Supplementary Table 1 | Summarizing device parameters of ST-OSCs reported in previous works.

Supplementary Table 2 | The summary of detailed device parameters of PM6:Qx-p-4Cl spin- and slot-die-coated opaque devices with different D:A ratios.

Supplementary Table 3 | The summary of detailed device parameters of PM6:Y6 spin- and slot-die-coated opaque devices with different D:A ratios.

Supplementary Table 4 | The summary of detailed device parameters of PM6:L8-BO spin- and slot-die-coated opaque devices with different D:A ratios.

Supplementary Table 5 | The summary of detailed device parameters of PM6:Qx-1 spin- and slot-die-coated opaque devices with different D:A ratios.

Supplementary Table 6 | The detailed parameters of semitransparent devices based on PM6:Qx-p-4Cl.

Supplementary Table 7 | The summary of calculated J_{SC} from EQE spectra for PM6:Qx-p-4Cl slot-die-coated semitransparent devices.

Supplementary Table 8 | Measuring the AVT values of spin-coated blends (1:1.5) and slot-die-coated blends (1:3) with different thicknesses.

Supplementary Table 9 | The detailed GIWAXS parameters of PM6:Qx-p-4Cl blends.

Supplementary Table 10 | Summarizing values of root mean square from AFM for PM6:Qx-p-4Cl blends.

Supplementary Table 11 | Summarizing the half-time of rising of hole transfer ($t_{half\text{-}time}$) of PM6:Qx-p-4Cl blends.

Supplementary Table 12 | The lifetimes fitted from the traces of LE state for PM6:Qx-p-4Cl blends.

Supplementary Table 13 | The lifetimes fitted from the traces of polaron state for PM6:Qx-p-4Cl blends.

Supplementary Table 14 | Fitting parameters for Qx-p-4Cl, Y6, L8-BO and Qx-1 fluence measurements.

Supplementary Table 15 | The Fitted L_D parameters.

Supplementary Table 16 | The lifetimes fitted from TRPL spectra.

Supplementary Table 17 | The detailed parameters of semitransparent modules.

Supplementary Table 18 | The viscosity of PM6:Qx-p-4Cl with different D:A ratios.

Supplementary Table 19 | Summarizing LUE of ST-modules reported in previous works.

Supplementary Table 20 | Summarizing CTM of PCE of opaque modules reported in previous works.

Supplementary Table 21 | Summarizing CTM of PCE and LUE of ST-modules reported in previous works.

Supplementary Table 22 | The summary of temperature inside the house model.

Supplementary Table 23 | The summary of weather data during the stability testing period.

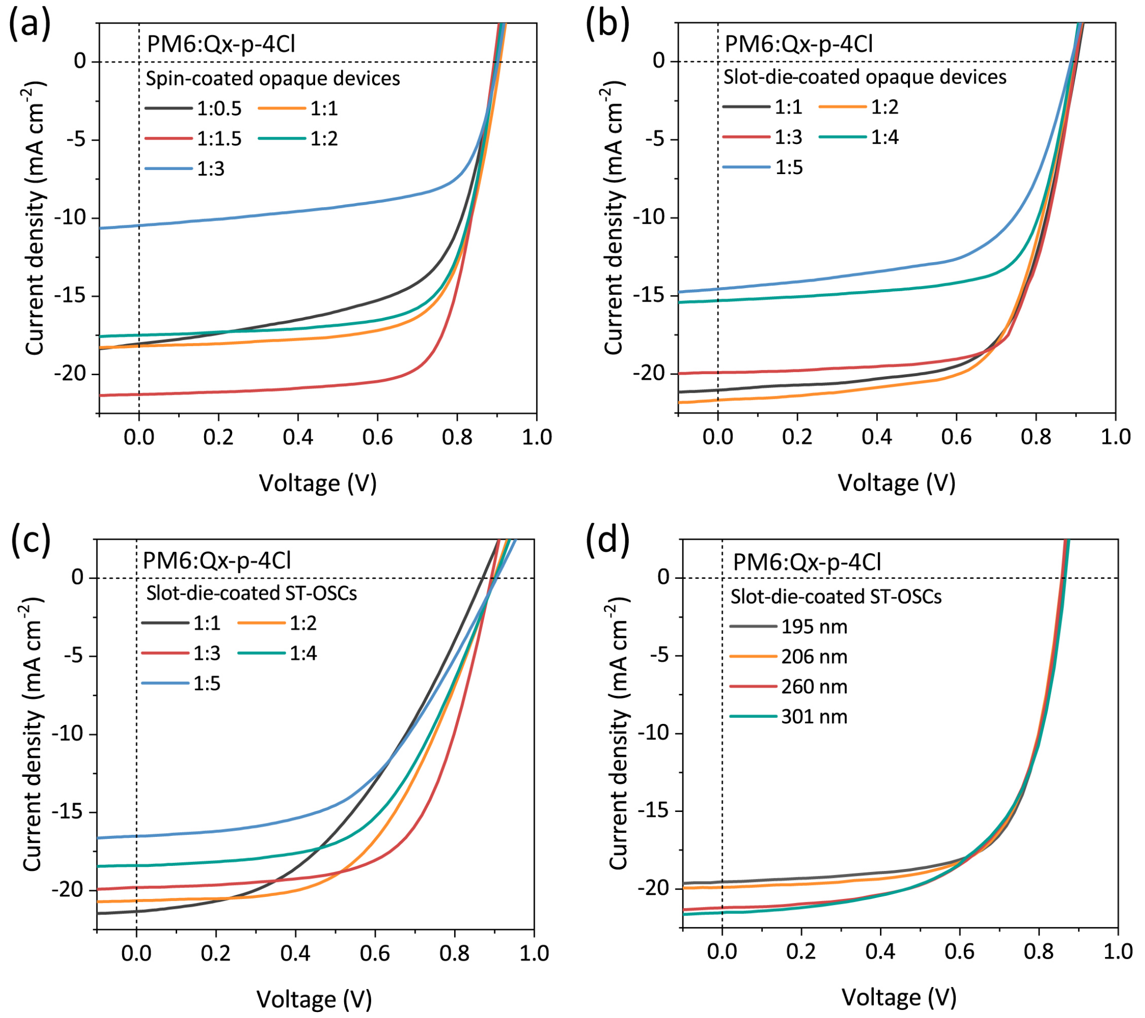

Supplementary Figure 1. The J-V curves of (a) spin-coated, (b) slot-die-coated PM6:Qx-p-4Cl opaque devices with different D:A ratios, (c) slot-die-coated PM6:Qx-p-4Cl ST-OSCs with different D:A ratios and (d) slot-die-coated PM6:Qx-p-4Cl ST-OSCs with different thickness.

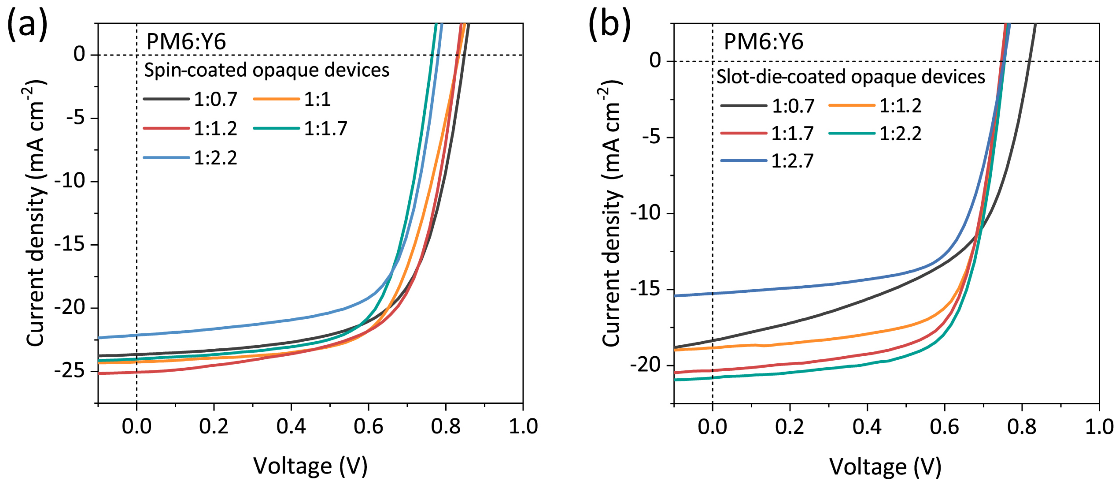

Supplementary Figure 2. The J-V curves of (a) spin-coated and (b) slot-die-coated PM6:Y6 opaque devices with different D:A ratios.

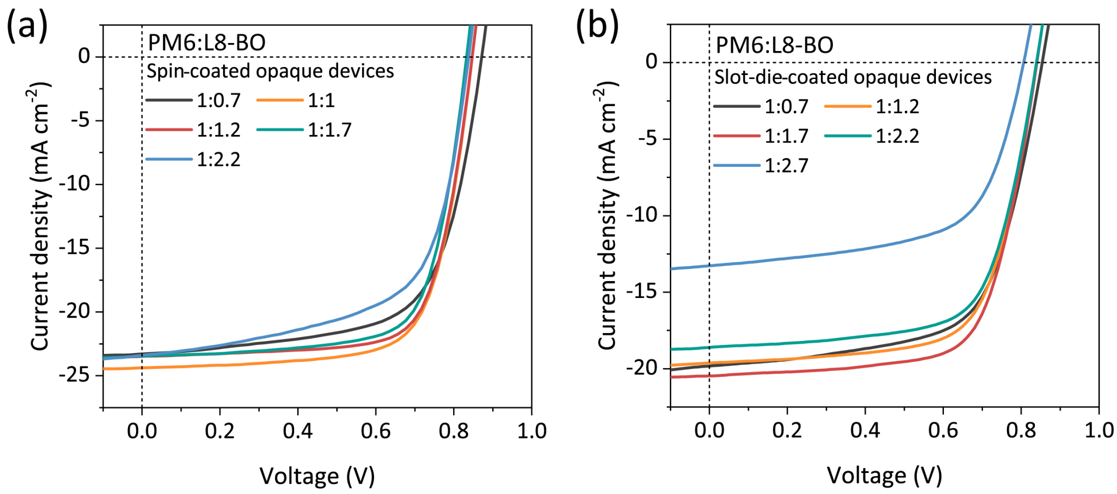

Supplementary Figure 3. The J-V curves of (a) spin-coated and (b) slot-die-coated PM6:L8-BO opaque devices with different D:A ratios.

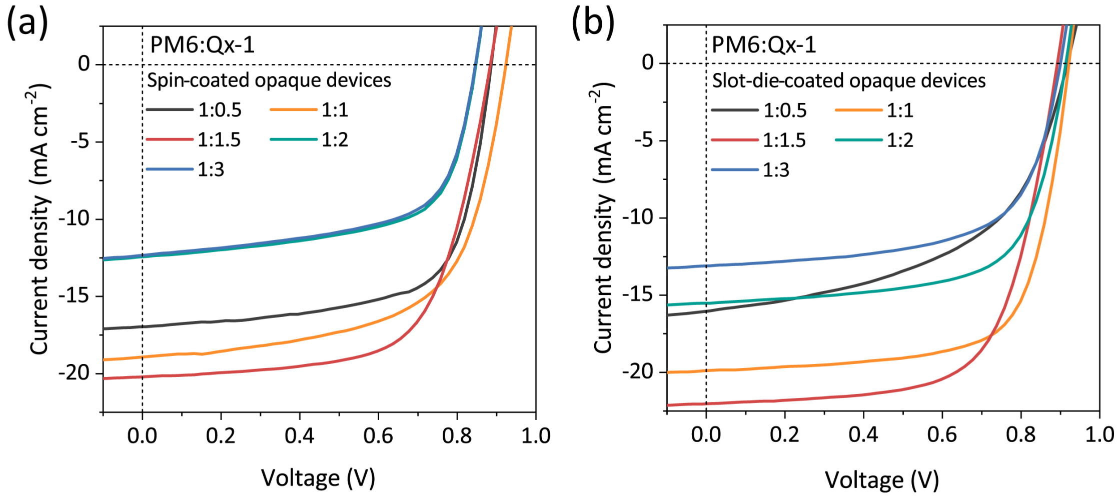

Supplementary Figure 4. The J-V curves of (a) spin-coated and (b) slot-die-coated PM6:Qx-1 opaque devices with different D:A ratios.

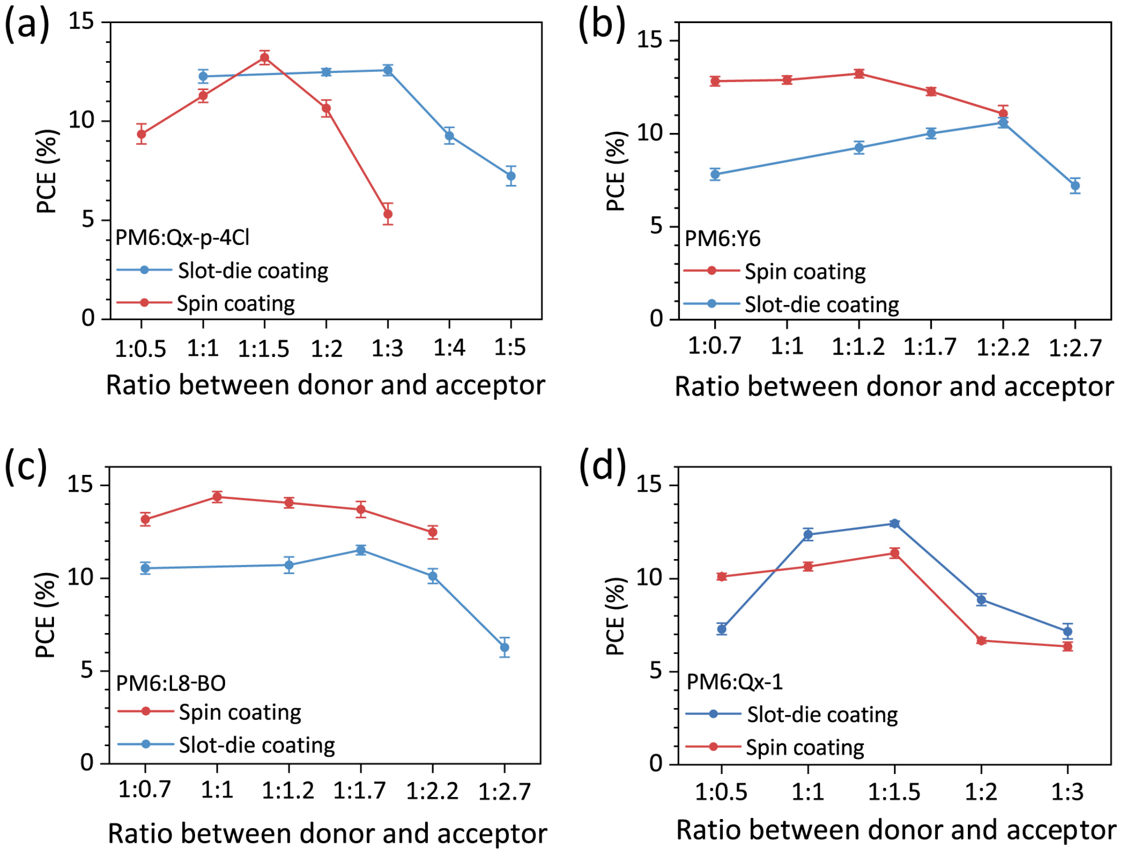

Supplementary Figure 5. PCE versus D:A ratios of (a) PM6:Qx-p-4Cl, (b) PM6:Y6, (c) PM6:L8-BO and (d) PM6:Qx-1. The error bars represent standard deviation.

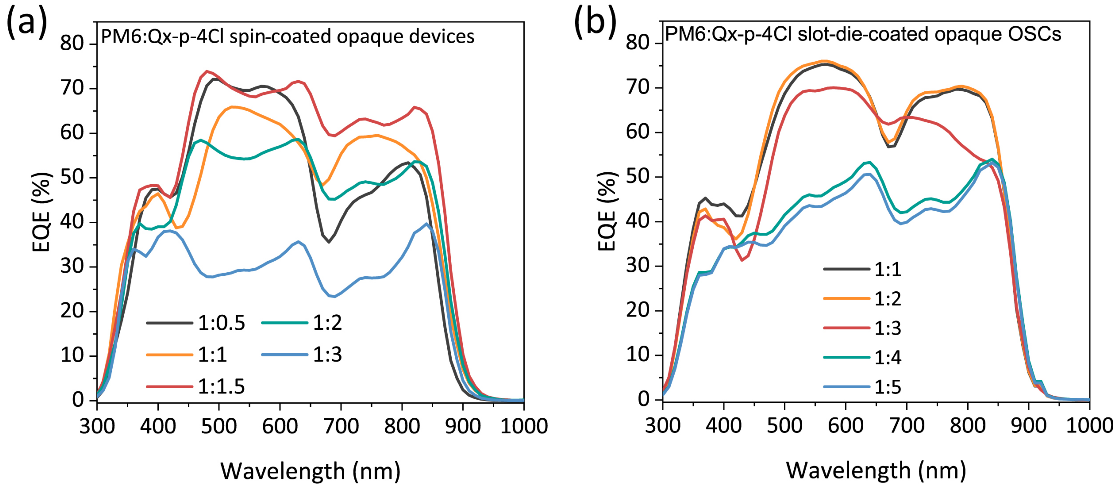

Supplementary Figure 6. The EQE spectra of (a) spin-coated and (b) slot-die-coated PM6:Qx-p-4Cl opaque devices with different D:A ratios.

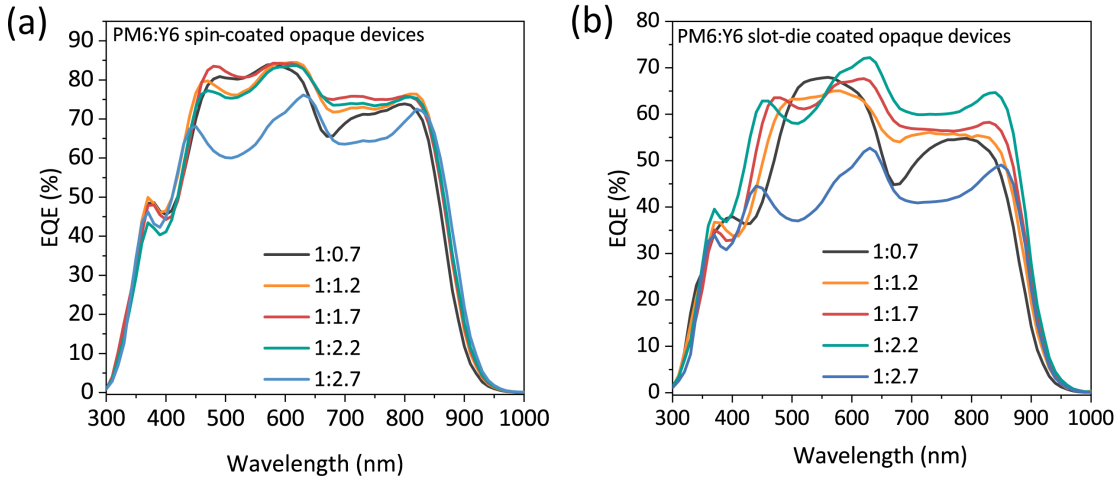

Supplementary Figure 7. The EQE spectra of (a) spin-coated and (b) slot-die-coated PM6:Y6 opaque devices with different D:A ratios.

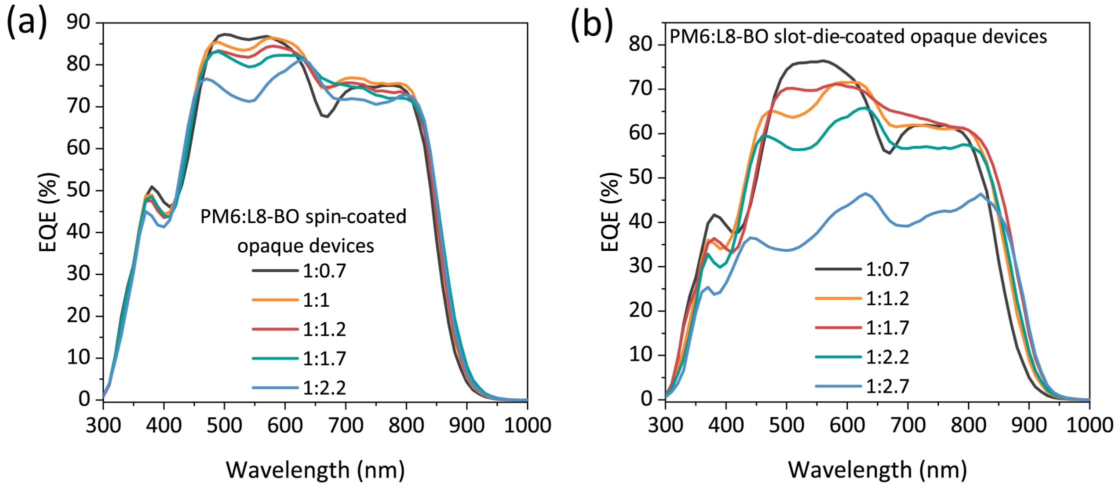

Supplementary Figure 8. The EQE spectra of (a) spin-coated and (b) slot-die-coated PM6:L8-BO opaque devices with different D:A ratios.

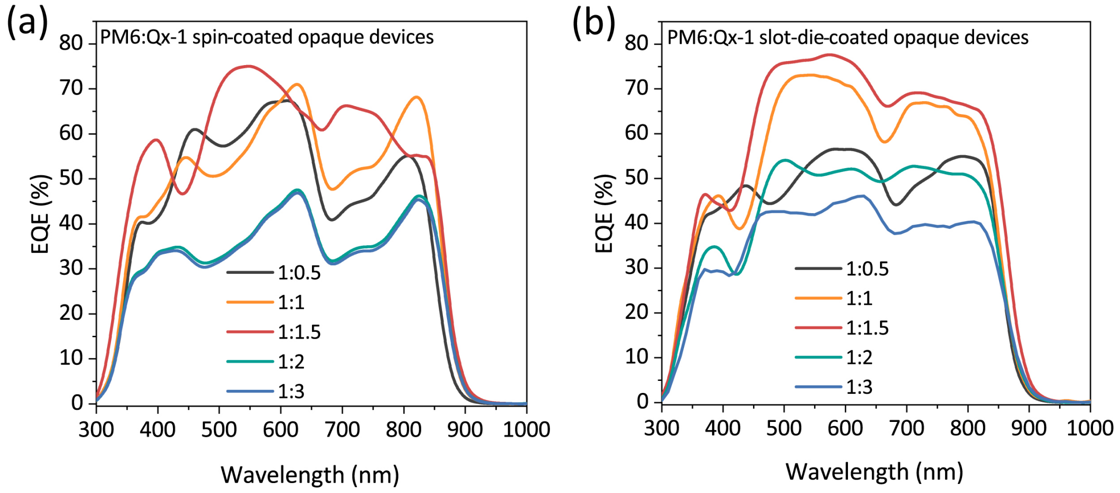

Supplementary Figure 9. The EQE spectra of (a) spin-coated and (b) slot-die-coated PM6:Qx-1 opaque devices with different D:A ratios.

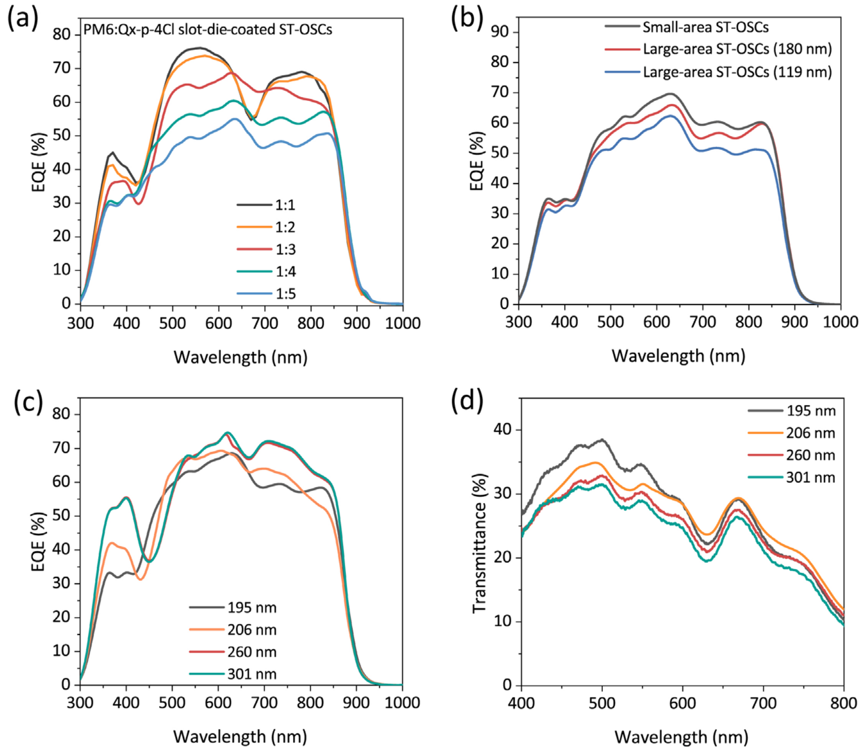

Supplementary Figure 10. The EQE spectra of slot-die-coated (a) PM6:P4Cl semitransparent devices with different D:A ratios, (b) MoO_3-modified PM6:Qx-p-4Cl (1:3) semitransparent devices with different device area and (c) larger active layer thickness. (d) The transmittance spectra of the MoO_3 ARC-modified large-area semitransparent device with different active layer thicknesses.

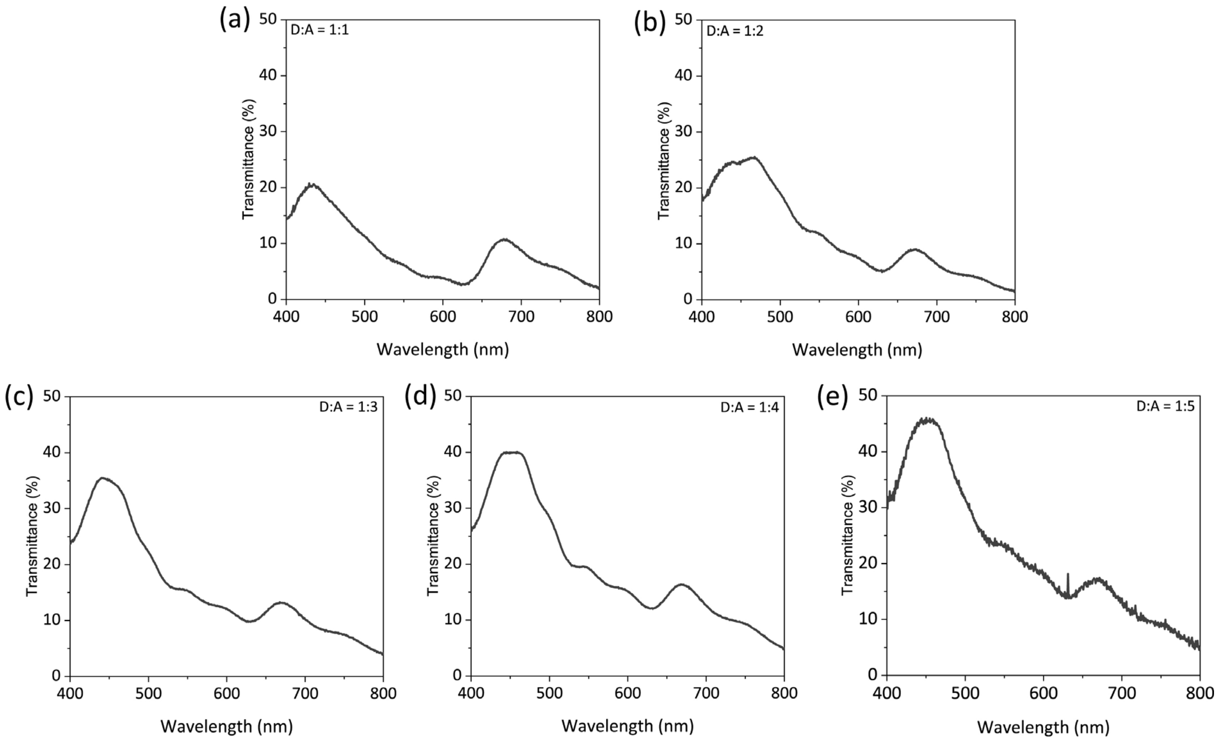

Supplementary Figure 11. The transmittance spectra of PM6:Qx-p-4Cl semitransparent

devices without MoO_3 ARC.

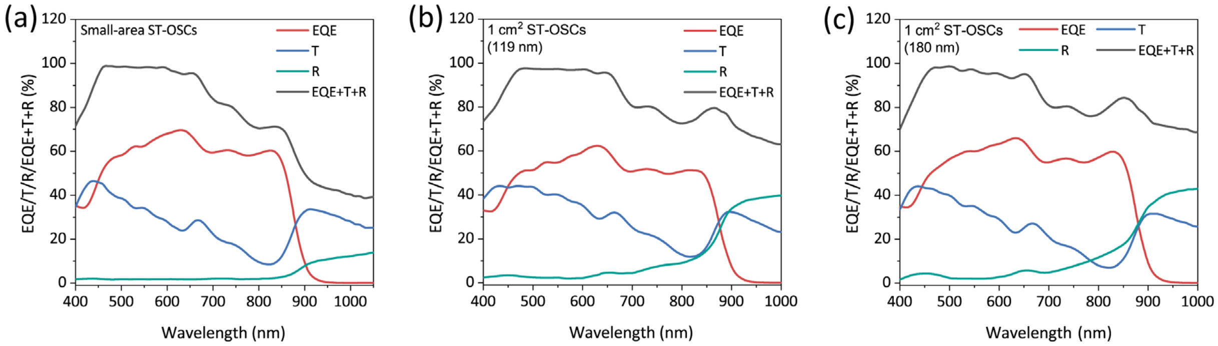

Supplementary Figure 12. The EQE, transmittance (T), reflection spectra (R) and EQE+T+R of MoO_3 ARC-modified (a) small-area semitransparent device, (b) 119 nm and (c) 180 nm large-area semitransparent device.

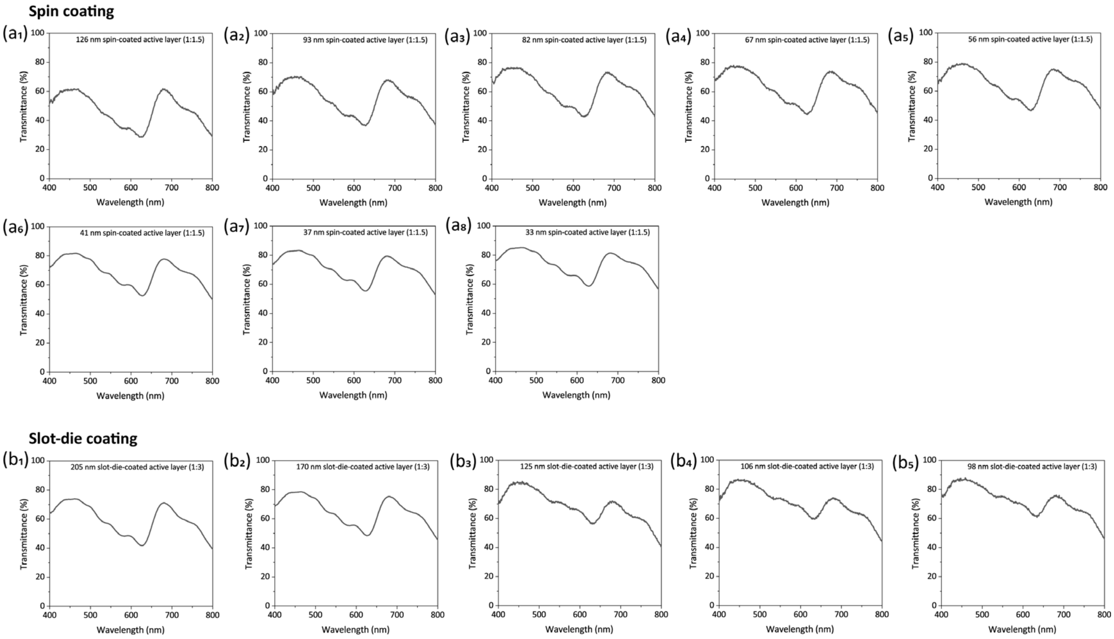

Supplementary Figure 13. The transmittance spectra of (a_{1-8}) PM6:Qx-p-4Cl (1:1.5) spin-coated active layer with different active layer and (b_{1-5}) PM6:Qx-p-4Cl (1:3) slot-die-coated active layer with different active layer thickness.

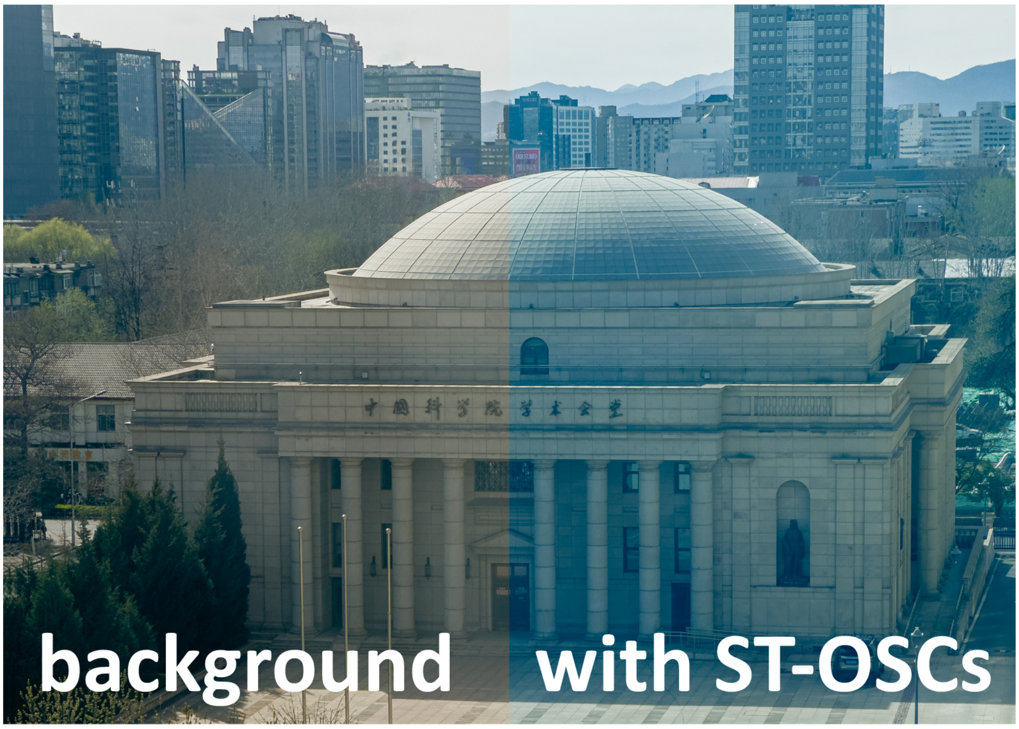

Supplementary Figure 14. Photographs showing semitransparent properties of ST-OSCs fabricated in this work.

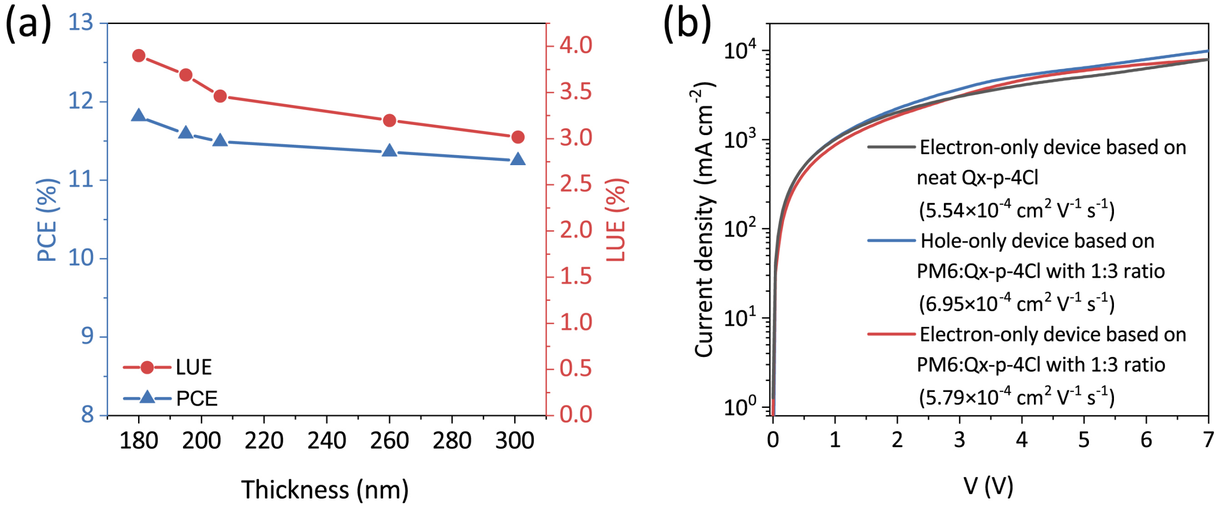

Supplementary Figure 15. (a) The PCE and LUE of large-area ST-OSCs with different thicknesses. (b) The J-V curves of electron-only device based on neat Qx-p-4Cl, hole- and electron-only devices based on PM6:Qx-p-4Cl (1:3).

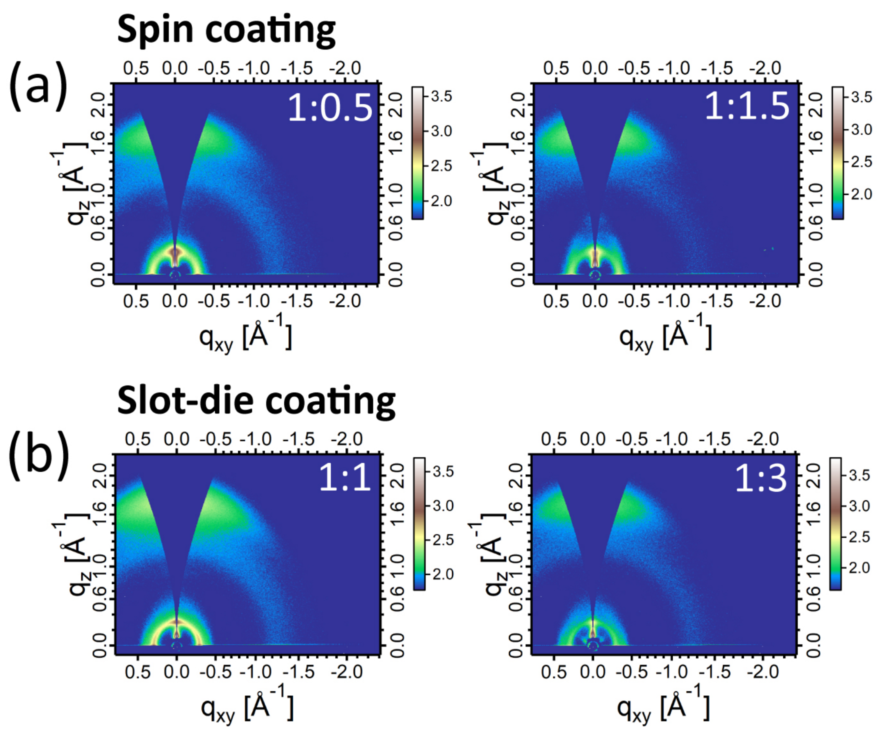

Supplementary Figure 16. 2D GIWAXS patterns (a) spin-coated and (b) slot-die-coated blends with different D:A ratios.

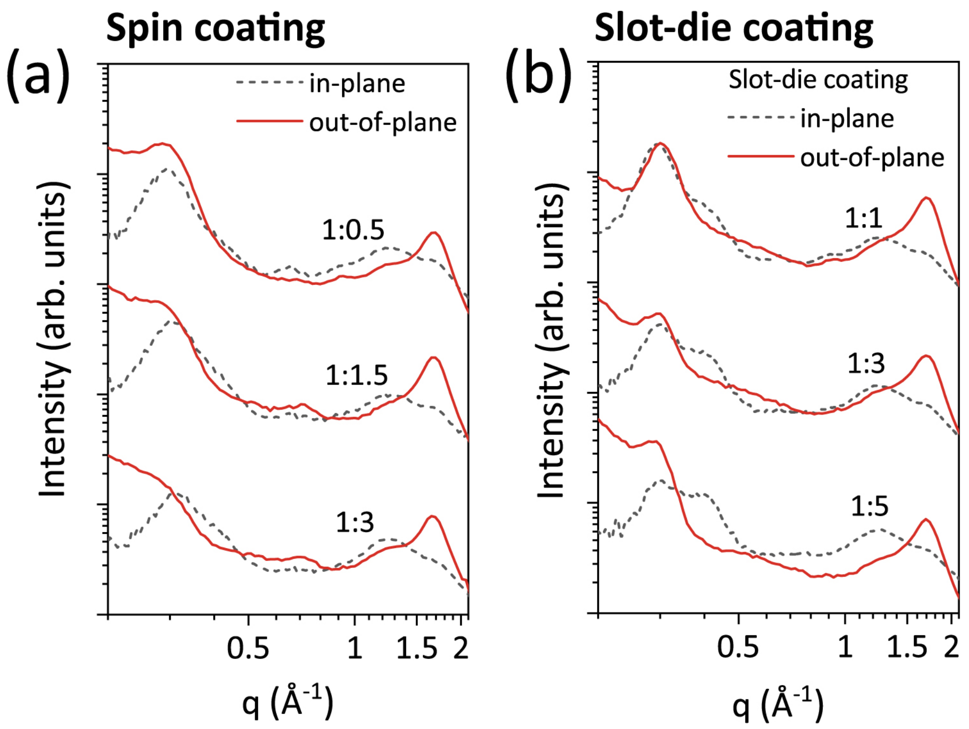

Supplementary Figure 17. 1D cut profiles along out-of-plane and in-plane directions of (**c**) spin-coated and (**d**) slot-die-coated films.

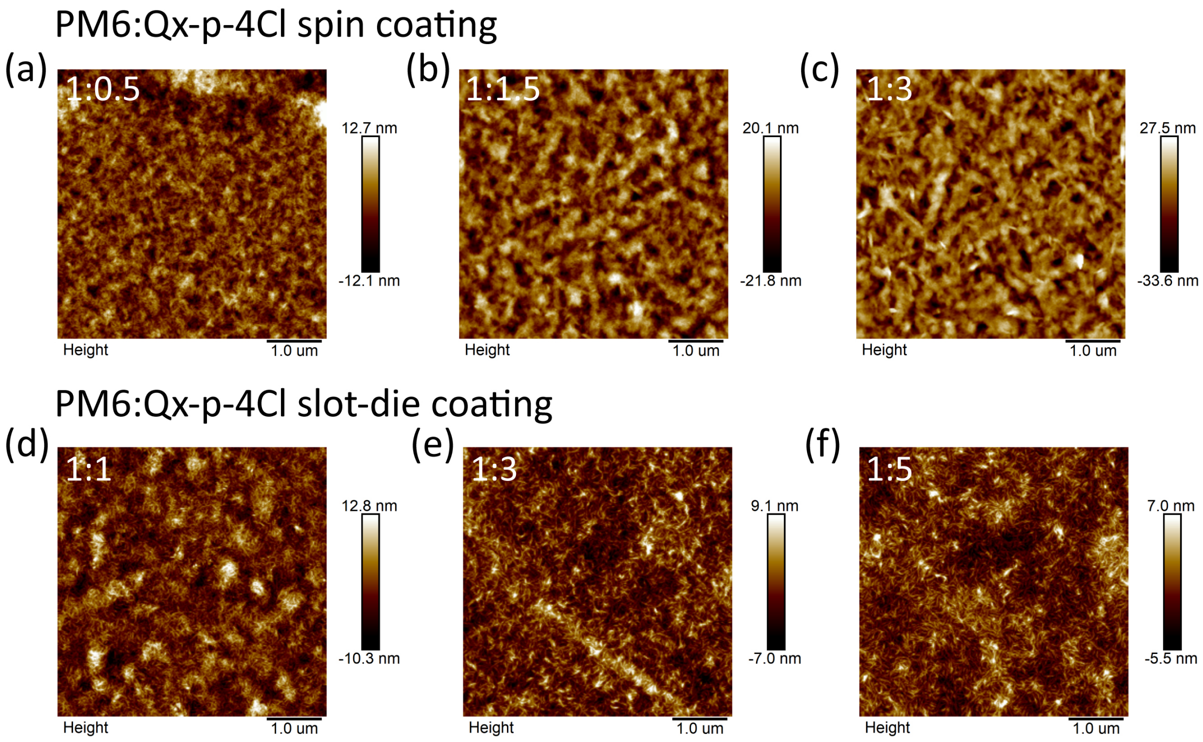

Supplementary Figure 18. The AFM height images of (a)-(c) spin-coated and (d)-(f) slot-die-coated PM6:Qx-p-4Cl films with different D:A ratios.

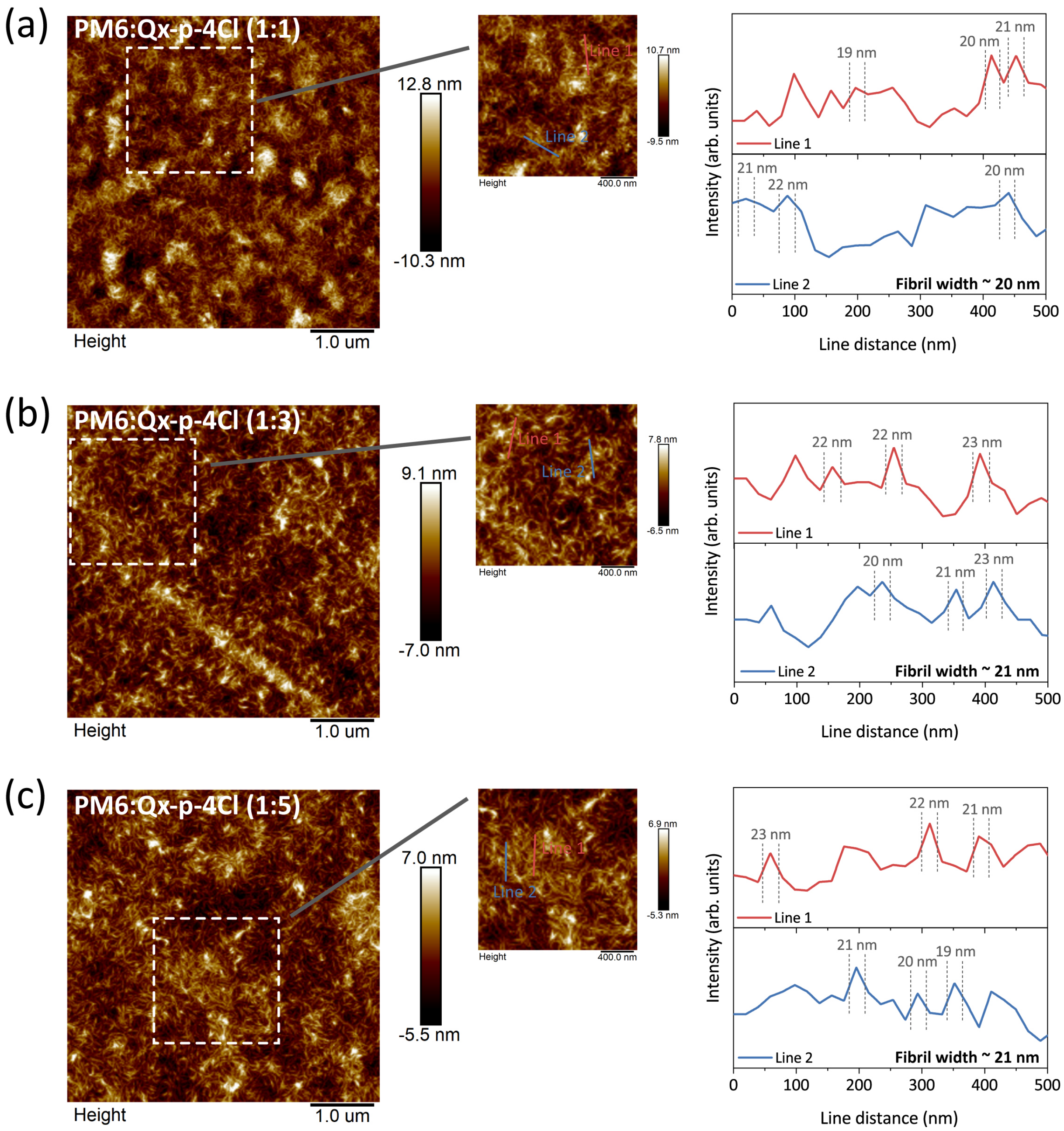

Supplementary Figure 19. The AFM height images and statistics of fibril width of slot-die-coated PM6:Qx-p-4Cl films with D:A ratios of (a) 1:1, (b) 1:3 and (c) 1:5.

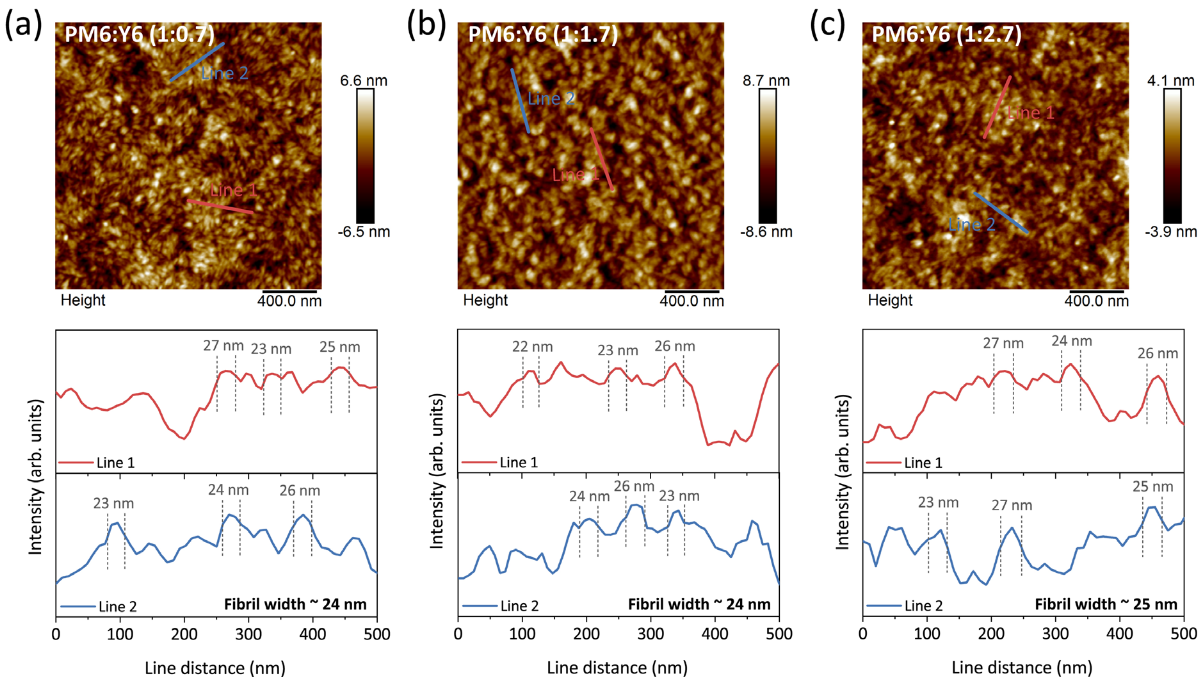

Supplementary Figure 20. The AFM height images and statistics of fibril width of slot-die-coated PM6:Y6 films with different D:A ratios.

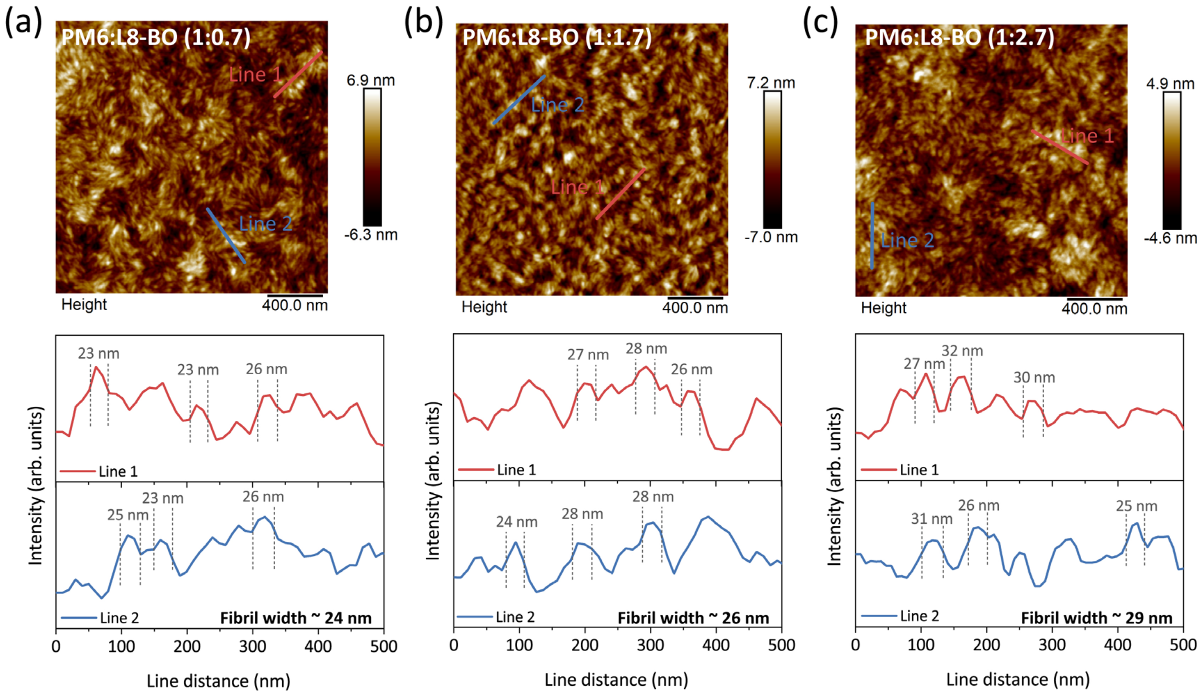

Supplementary Figure 21. The AFM height images and statistics of fibril width of slot-die-coated PM6:L8-BO films with different D:A ratios.

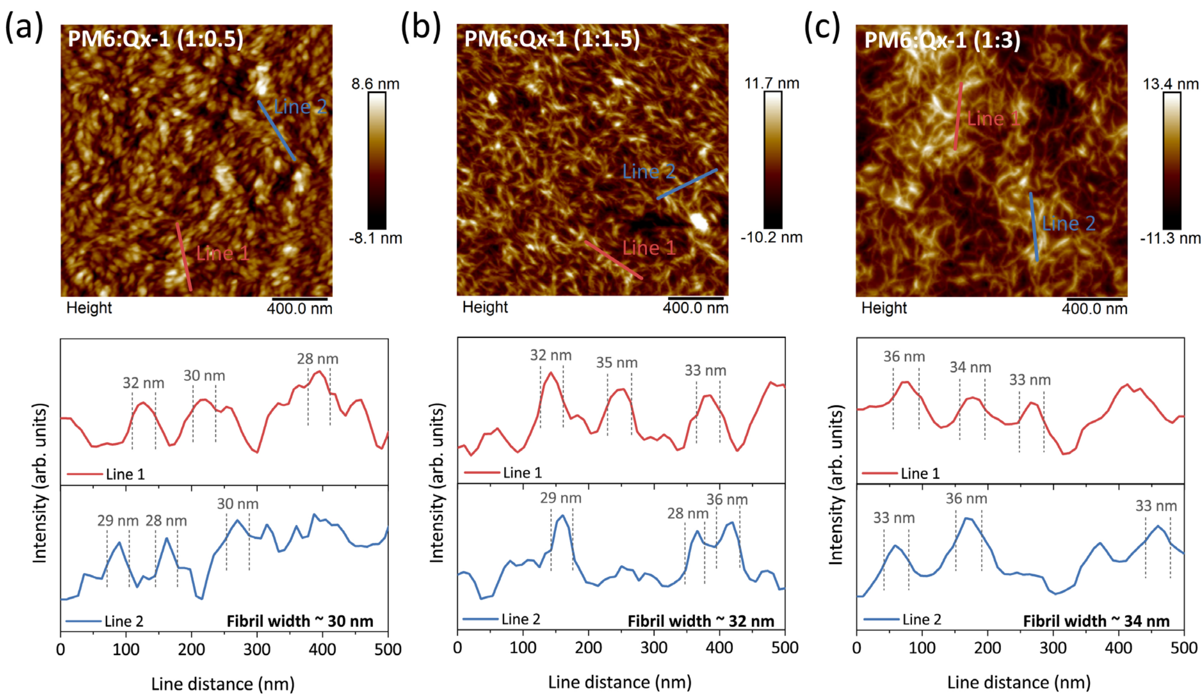

Supplementary Figure 22. The AFM height images and statistics of fibril width of slot-die-coated PM6:Qx-1 films with different D:A ratios.

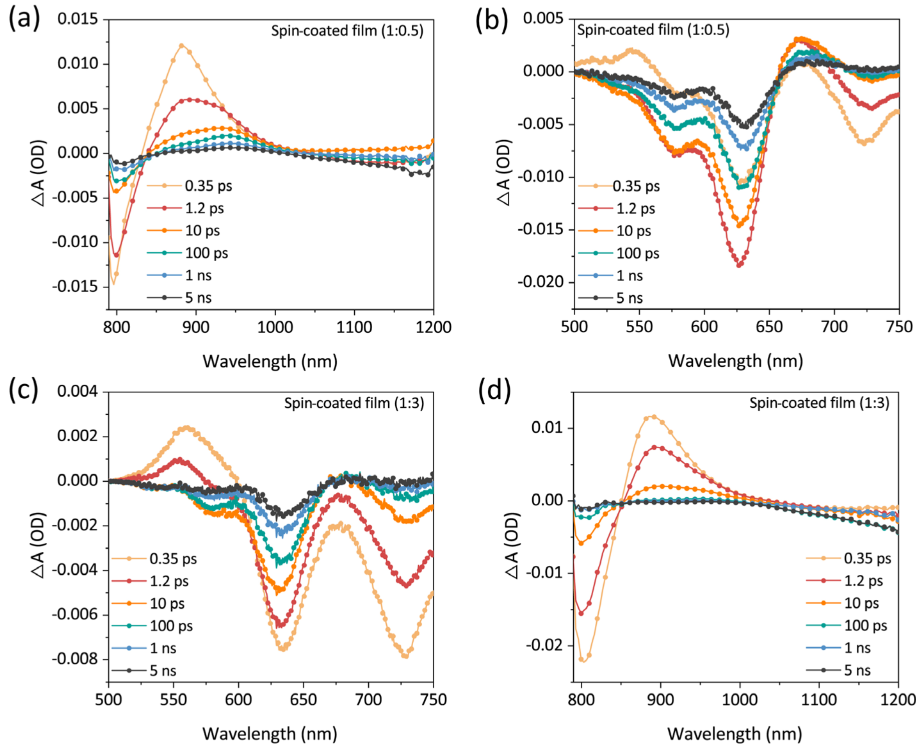

Supplementary Figure 23. TA spectra for spin-coated (a)-(b) PM6:Qx-p-4Cl with D:A ratio of 1:0.5 and (c)-(d) 1:3.

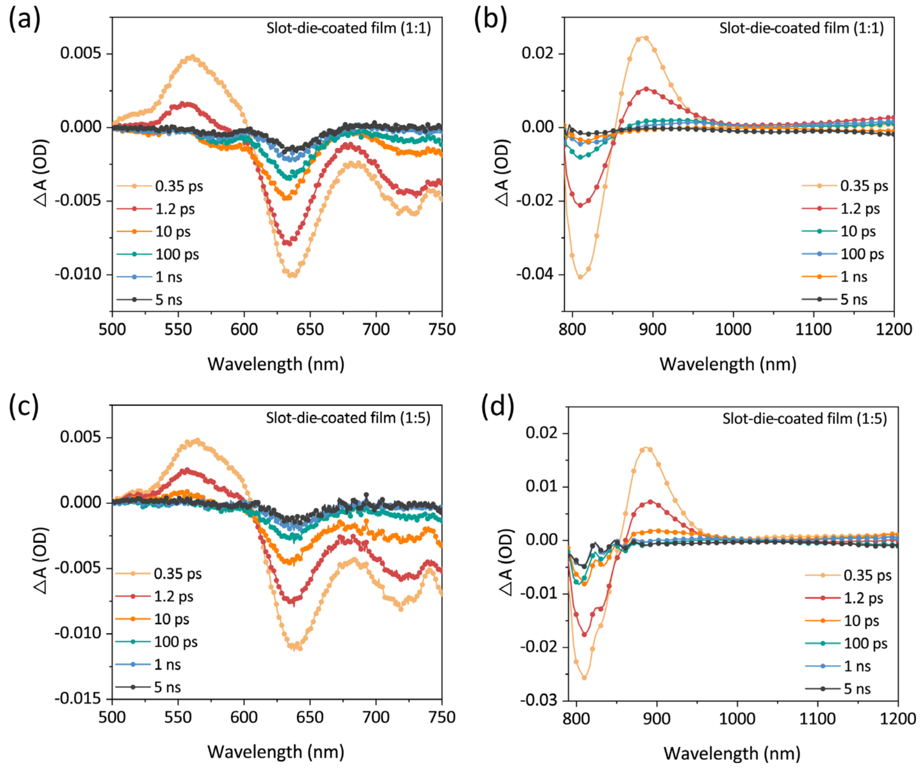

Supplementary Figure 24. TA spectra for slot-die-coated (a)-(b) PM6:Qx-p-4Cl with D:A ratio of 1:1 and (c)-(d) 1:5.

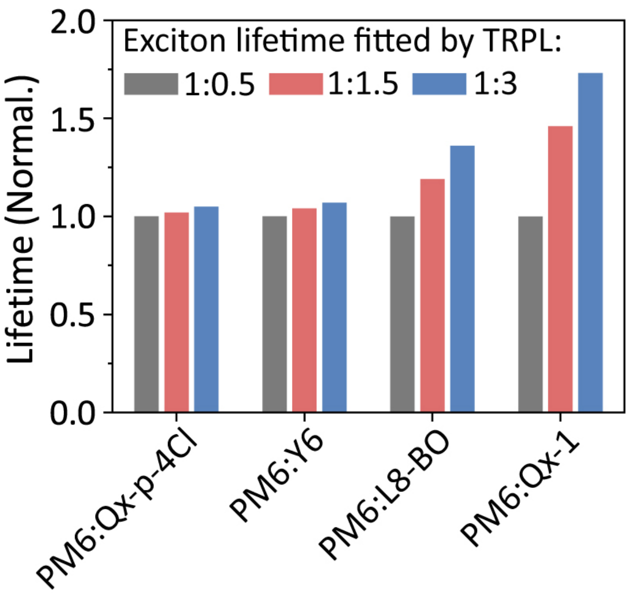

Supplementary Figure 25. The summary of exciton lifetimes normalized against 1:0.5 films.

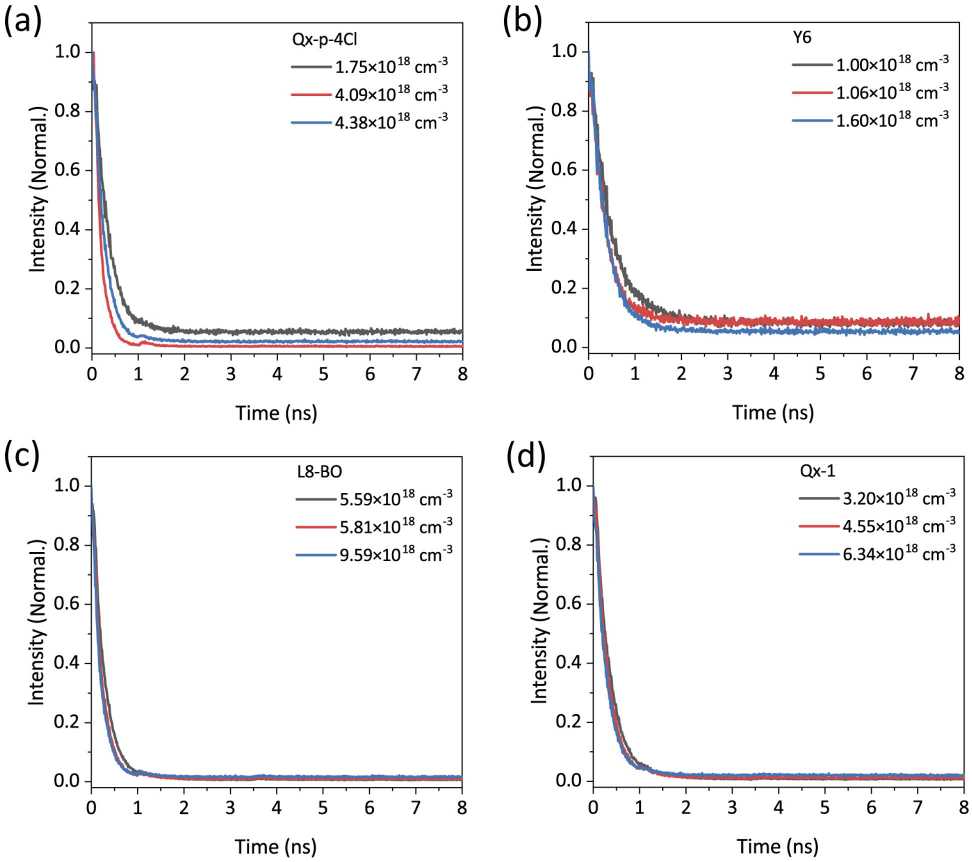

Supplementary Figure 26. Fluence dependent PL decays for (a) Qx-p-4Cl, (b) Y6, (c) L8-BO and (d) Qx-1.

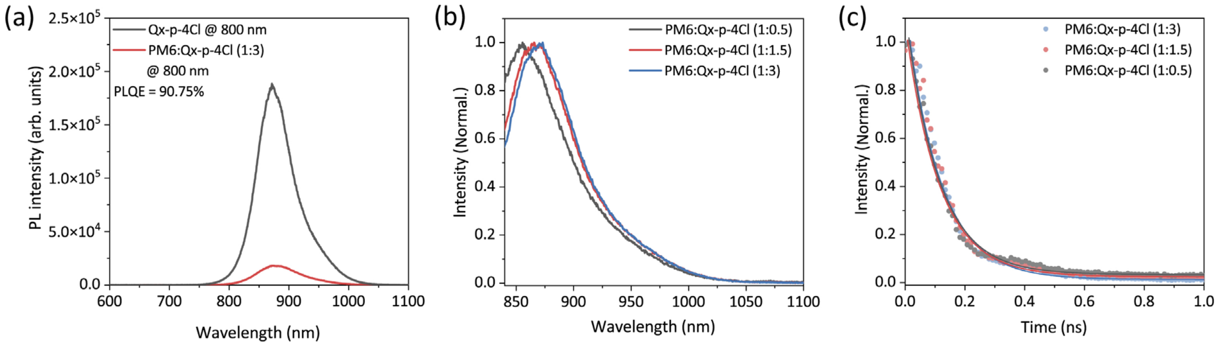

Supplementary Figure 27. (a) The PL spectra of neat Qx-p-4Cl and PM6:Qx-p-4Cl (1:3) blend for calculation of PL quenching efficiency (PLQE). The (b) PL and (c) TRPL spectra of PM6:Qx-p-4Cl blends with different D:A ratios.

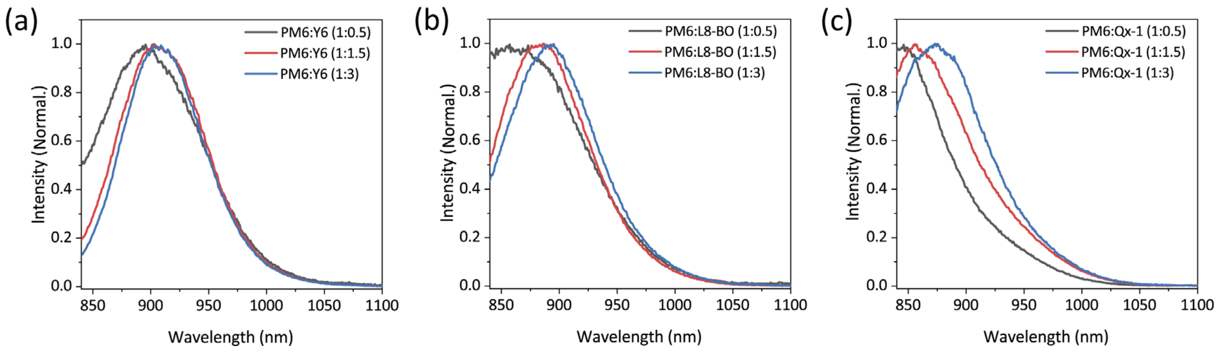

Supplementary Figure 28. PL spectra of (a) PM6:Y6, (b) PM6:L8-BO and (c) PM6:Qx-1 with different D:A ratios.

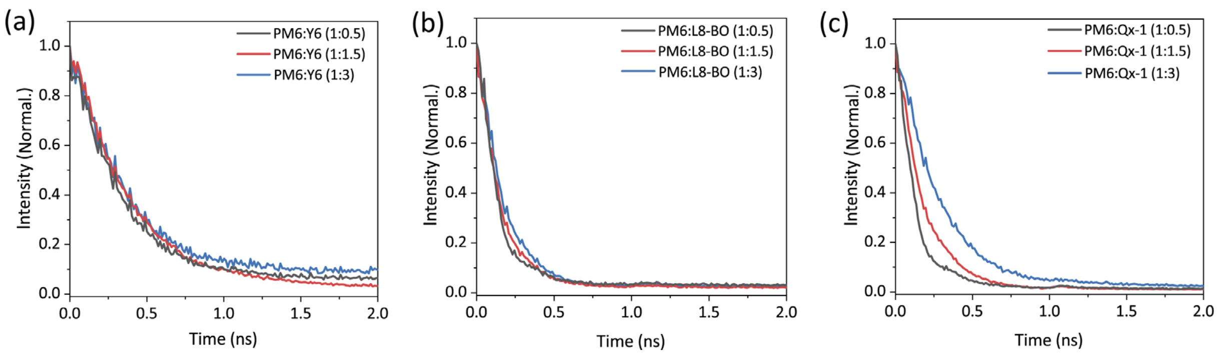

Supplementary Figure 29. TRPL spectra of (a) PM6:Y6, (b) PM6:L8-BO and (c) PM6:Qx-1 with different D:A ratios.

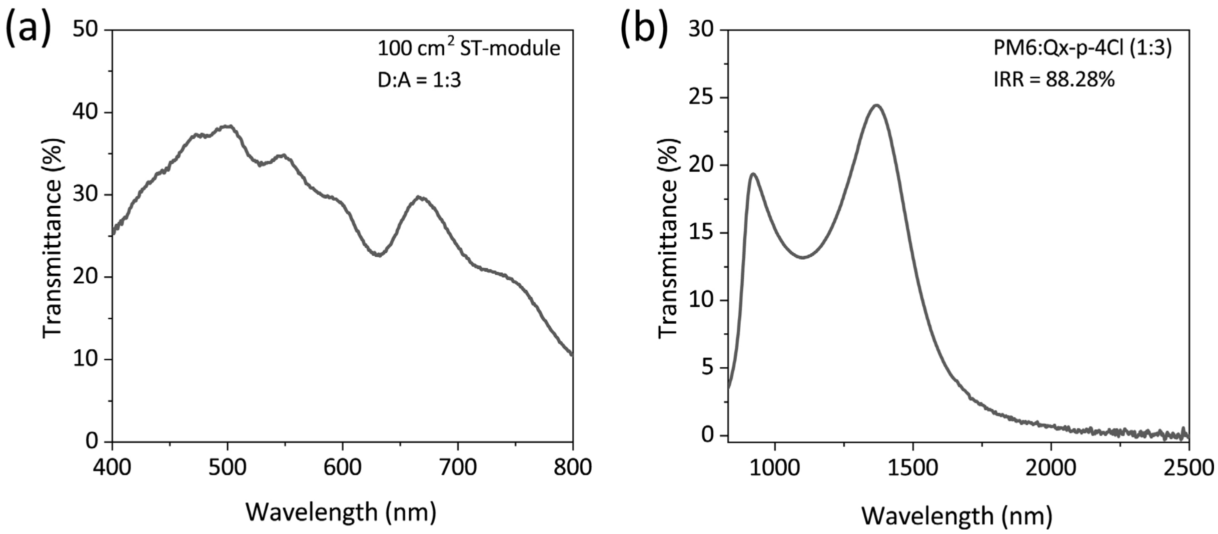

Supplementary Figure 30. (a) The transmittance spectrum of 100 cm^2 ST-modules in the visible range (400-800 nm). (b) The transmittance spectrum of PM6:Qx-p-4Cl (D:A = 1:3) in the near-infrared range (830-2500 nm) and the calculation of infrared radiation rejection (IRR).

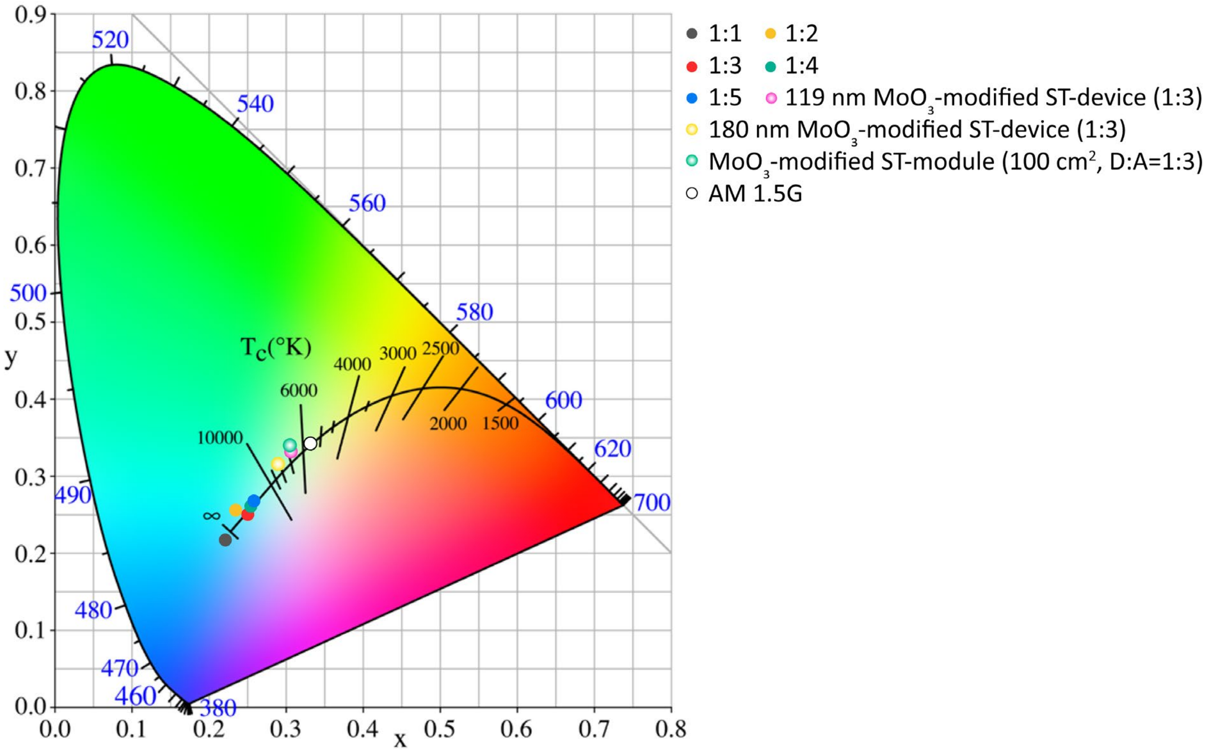

Supplementary Figure 31. CIE-1931 chromaticity diagrams of PM6:Qx-p-4Cl ST-OSCs.

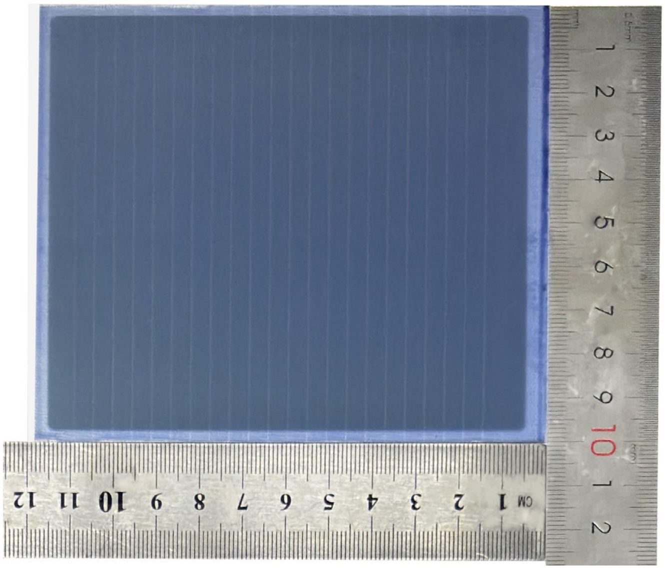

Supplementary Figure 32. The detailed size of 100 cm^2 ST-module fabricated in this work.

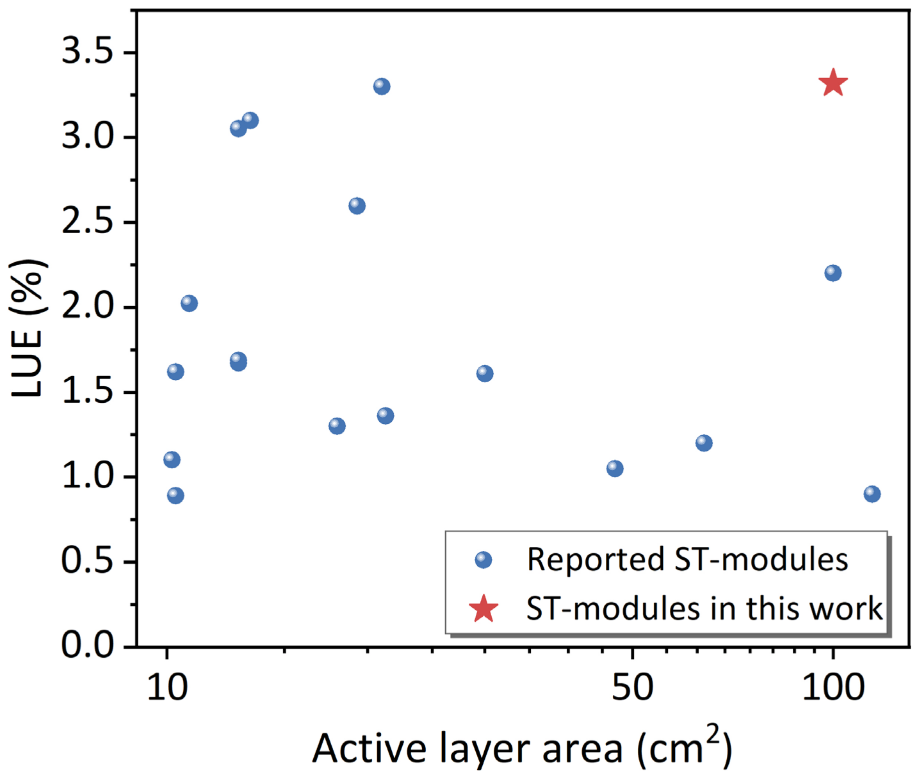

Supplementary Figure 33. The summary of LUE versus active layer area of reported ST-modules.

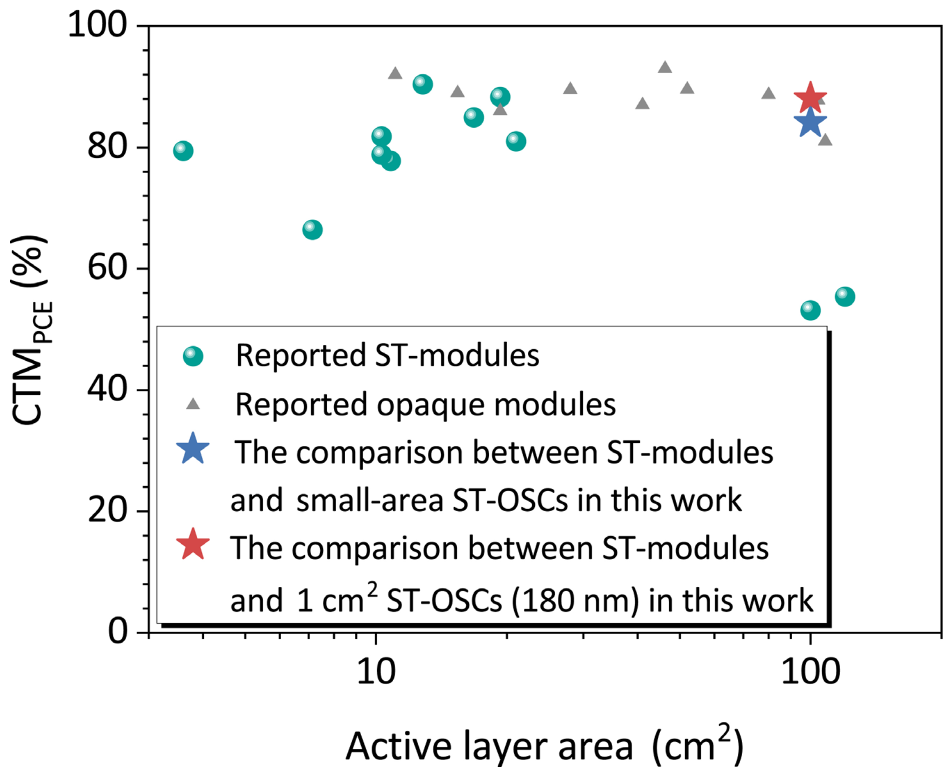

Supplementary Figure 34. The summary of the CTM of PCE versus the active layer area of reported large-area opaque modules and ST-modules.

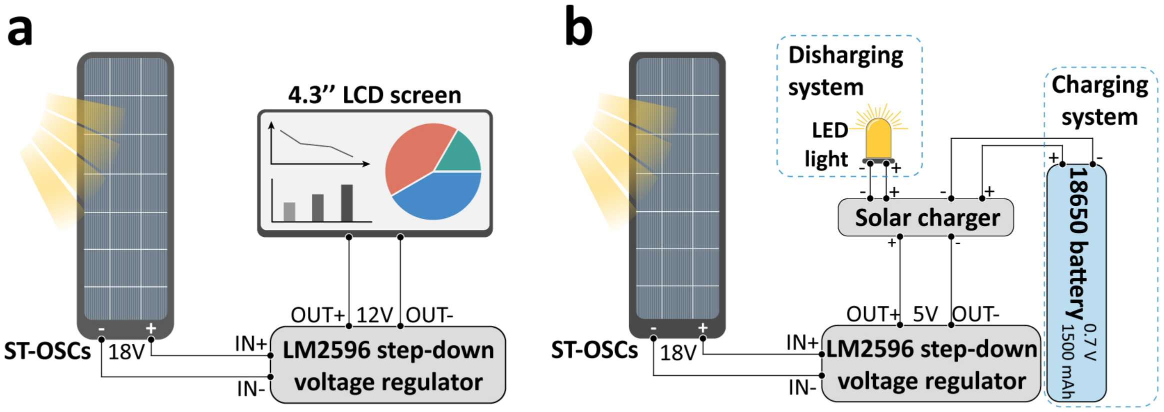

Supplementary Figure 35. The block diagrams of **(e)** LCD drive system and **(f)** energy storage system.

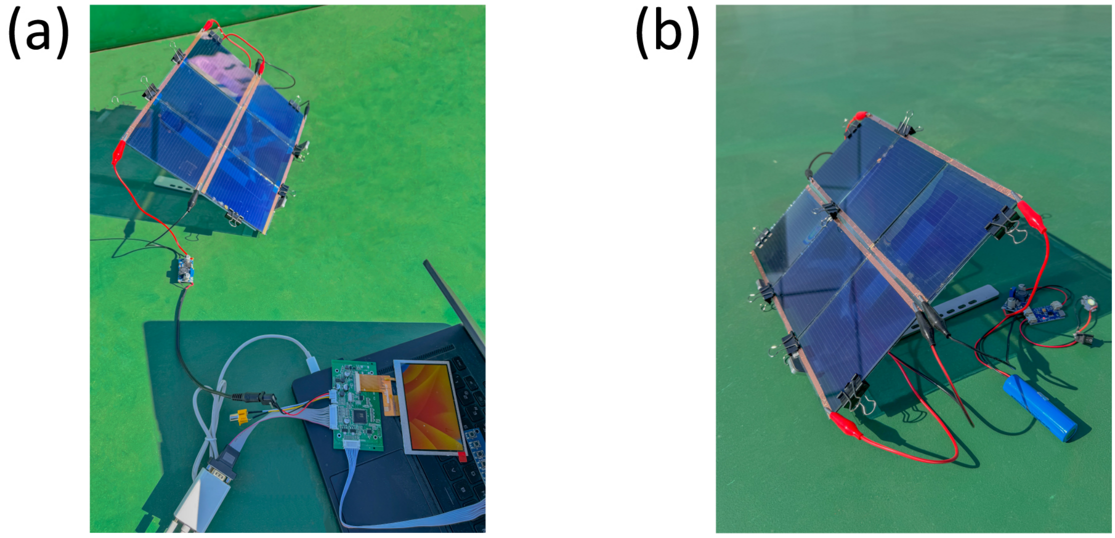

Supplementary Figure 36. The photographs of powering (a) LCD screen and (b) 18650 battery.

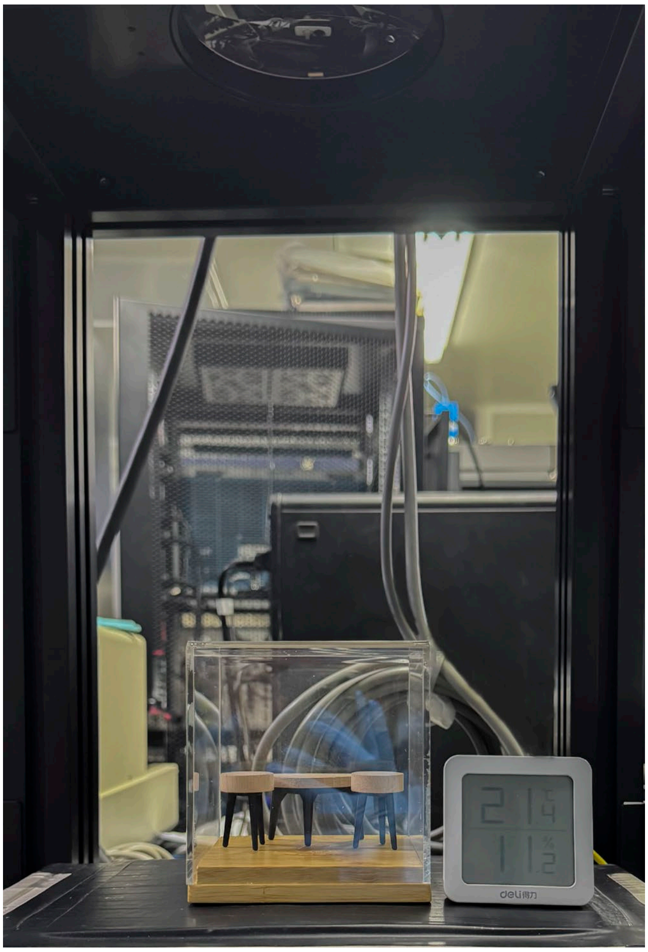

Supplementary Figure 37. The photograph of ambient temperature.

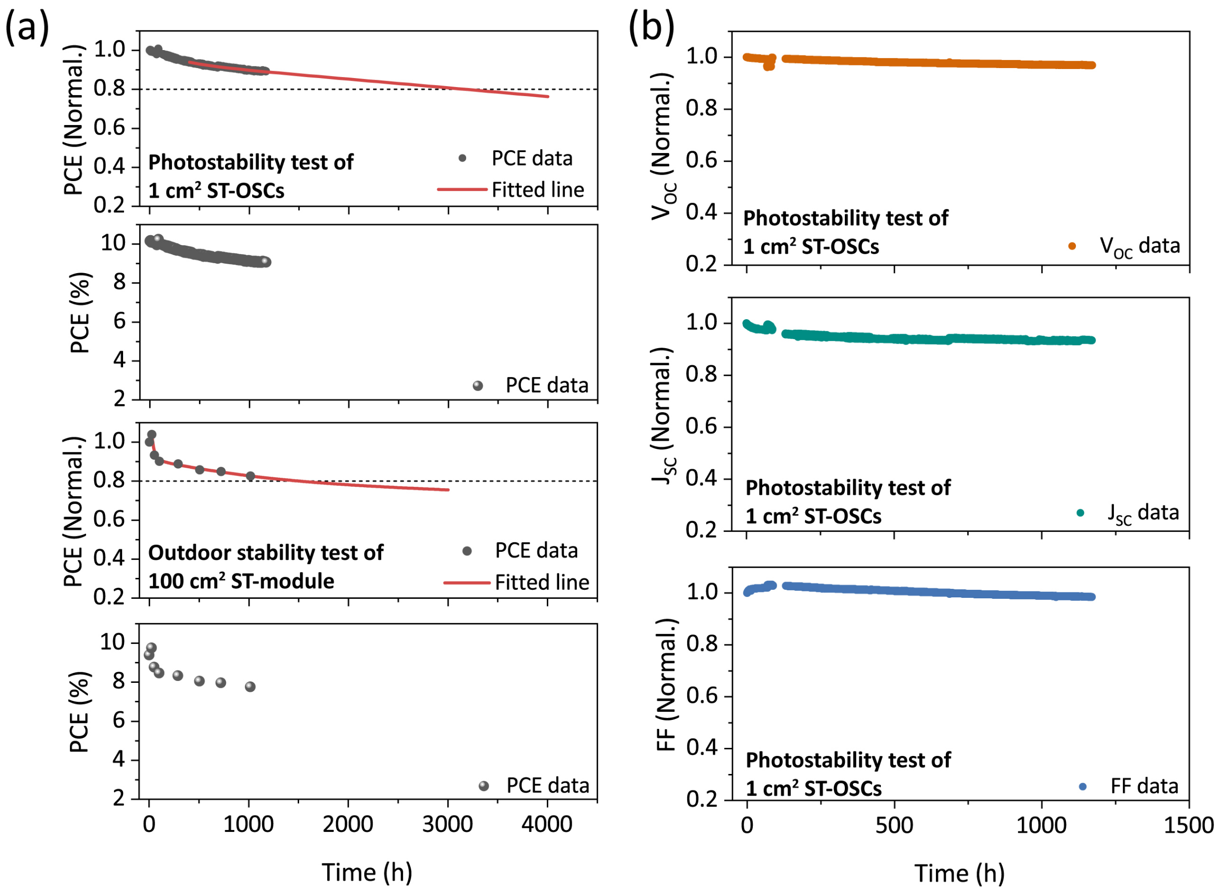

Supplementary Figure 38. (a) Normalized PCE with testing time for 1 cm^2 ST-OSCs (D:A = 1:3) and 100 cm^2 ST-module. (b) Normalized V_{OC}, J_{SC}, and FF with light-soaking time for 1 cm^2 ST-OSCs (D:A = 1:3).

Supplementary Table 1. The device parameters of reported works in Figure 1h.

Num.	Area (cm^2)	Thickness (nm)	PCE (%)	AVT (%)	LUE (%)	Reference
1	1.25	100	8.05	16.49	1.33	[1]
2	1.25	100	6.67	21.36	1.42	[1]
3	3.6	100	9.74	21.2	2.06	[2]
4	1.05	75	13.93	19.93	2.78	[3]
5	1.05	75	12.09	32.47	3.93	[3]
6	10.8	100	9.54	21.2	2.02	[2]
7	10.17	185	9.75	11.3	1.10	[4]
8	12.8	80	7.50	22.3	1.67	[5]
9	12.8	80	7.30	41.8	3.05	[5]
10	12.8	80	7.40	22.8	1.69	[5]
11	19.3	70	11.65	22.3	2.60	[6]
12	21.0	90	10.20	32.8	3.35	[7]
13	100.0	90	6.70	32.9	2.20	[7]

Supplementary Table 2. The detailed device parameters of PM6:Qx-p-4Cl opaque devices.

Processing methods	D:A ratios	V_{OC} (V)	J_{SC} (mA cm^{-2})	J_{cal} (mA cm^{-2})	FF (%)	PCE (%)
Spin coating	1:0.5	0.90	18.04	17.30	61.20	9.91 (9.36±0.51)
	1:1	0.91	18.19	17.86	69.70	11.50 (11.29±0.33)
	1:1.5	0.89	21.29	20.83	72.61	13.80 (13.21±0.36)
	1:2	0.90	17.49	16.65	70.81	11.12 (10.65±0.43)
	1:3	0.90	10.48	10.05	64.56	6.10 (5.32±0.55)
Slot-die coating	1:1	0.90	21.03	20.66	65.92	12.50 (12.27±0.34)
	1:2	0.90	21.68	20.84	65.45	12.73 (12.48±0.18)
	1:3	0.90	19.91	18.98	71.44	12.79 (12.58±0.27)
	1:4	0.89	15.31	14.77	70.07	9.56 (9.27±0.42)
	1:5	0.89	14.56	14.12	60.43	7.82 (7.24±0.54)

Supplementary Table 3. The detailed device parameters of PM6:Y6 opaque devices.

Processing methods	D:A ratios	V_{OC} (V)	J_{SC} (mA cm^{-2})	J_{cal} (mA cm^{-2})	FF (%)	PCE (%)
Spin coating	1:0.7	0.85	23.63	23.17	65.10	13.04 (12.83±0.25)
	1:1	0.83	24.25	24.07	65.08	13.15 (12.90±0.22)
	1:1.2	0.83	24.97	24.35	65.01	13.47 (13.23±0.22)
	1:1.7	0.76	23.98	23.90	67.80	12.32 (12.27±0.20)
	1:2.2	0.78	22.13	21.59	67.27	11.62 (11.08±0.44)
Slot-die coating	1:0.7	0.81	18.47	17.65	55.33	8.23 (7.95±0.24)
	1:1.2	0.79	22.52	22.00	64.35	11.52 (10.85±0.45)
	1:1.7	0.79	21.80	20.93	68.48	11.79 (11.49±0.34)
	1:2.2	0.77	15.62	14.89	66.87	8.08 (7.57±0.49)
	1:2.7	0.75	15.28	14.57	66.29	7.62 (7.21±0.41)

Supplementary Table 4. The detailed device parameters of PM6:L8-BO opaque devices.

Processing methods	D:A ratios	V_{OC} (V)	J_{SC} (mA cm^{-2})	J_{cal} (mA cm^{-2})	FF (%)	PCE (%)
Spin coating	1:0.7	0.87	23.20	22.98	66.04	13.36 (13.17±0.36)
	1:1	0.85	24.35	23.60	71.30	14.71 (14.38±0.30)
	1:1.2	0.85	23.46	23.22	72.64	14.44 (14.07±0.27)
	1:1.7	0.83	23.46	23.23	71.54	13.98 (13.70±0.43)
	1:2.2	0.84	23.35	22.29	62.29	12.20 (12.43±0.34)
Slot-die coating	1:0.7	0.85	19.39	19.20	64.56	10.93 (10.54±0.32)
	1:1.2	0.84	19.45	19.44	67.96	11.20 (10.71±0.44)
	1:1.7	0.84	20.24	20.06	69.03	11.87 (11.52±0.24)
	1:2.2	0.84	18.61	18.06	67.90	10.60 (10.12±0.40)
	1:2.7	0.81	13.28	13.20	62.12	6.65 (6.28±0.53)

Supplementary Table 5. The detailed device parameters of PM6:Qx-1 opaque devices.

Processing methods	D:A ratios	V_{OC} (V)	J_{SC} (mA cm^{-2})	J_{cal} (mA cm^{-2})	FF (%)	PCE (%)
Spin coating	1:0.5	0.89	16.94	16.66	67.82	10.18 (10.11±0.1)
	1:1	0.92	18.90	17.99	62.02	10.83 (10.64±0.13)
	1:1.5	0.88	20.20	20.04	65.48	11.69 (11.36±0.26)
	1:2	0.85	12.42	12.05	63.90	6.72 (6.67±0.05)
	1:3	0.85	12.29	11.81	63.02	6.56 (6.35±0.23)
Slot-die coating	1:0.5	0.92	16.00	15.73	52.37	7.69 (7.30±0.31)
	1:1	0.92	19.88	19.58	70.01	12.83 (12.37±0.32)
	1:1.5	0.89	22.01	21.46	66.21	13.00 (12.87±0.20)
	1:2	0.91	15.49	15.22	66.80	9.46 (8.87±0.32)
	1:3	0.90	13.09	12.66	63.36	7.47 (7.17±0.41)

Supplementary Table 6. The detailed parameters of semitransparent devices based on PM6:Qx-p-4Cl.

Device Areas	ARC-modification	D:A ratios	V_{OC} (V)	J_{SC} (mA cm^{-2})	FF (%)	PCE_{max} (%) (PCE_{avg})	AVT (%)	LUE (%)
1 cm^2	w/o	1:1	0.87	21.34	43.86	8.14 (7.78±0.23)	6.41	0.52
1 cm^2	w/o	1:2	0.90	20.65	54.19	10.06 (9.58±0.31)	11.45	1.15
1 cm^2	w/o	1:3	0.89	19.80	63.73	11.25 (10.92±0.30)	15.43	1.74
1 cm^2	w/o	1:4	0.90	17.73	55.34	9.17 (8.91±0.25)	19.20	1.76
1 cm^2	w/o	1:5	0.90	15.52	50.93	7.61 (7.35±0.39)	22.83	1.74
1 cm^2 (119 nm)	with	1:3	0.90	17.48	67.35	10.57 (10.36±0.23)	38.21	4.04
1 cm^2 (180 nm)	with	1:3	0.89	18.86	70.32	11.81 (11.52±0.22)	33.01	3.90
1 cm^2 (195 nm)	with	1:3	0.86	19.54	68.90	11.59 (11.28±0.21)	31.83	3.69
1 cm^2 (206 nm)	with	1:3	0.86	19.86	67.43	11.49 (11.27±0.18)	30.09	3.46
1 cm^2	with	1:3	0.86	21.19	62.47	11.36	28.15	3.20

(260 nm)						(11.12±0.25)		
1 cm^2 (301 nm)	with	1:3	0.86	21.52	60.39	11.25 (10.96±0.34)	26.82	3.02
0.0256 cm^2	with	1:3	0.87	19.85	71.66	12.37 (12.00±0.28)	32.36	4.00

Supplementary Table 7. The calculated J_{SC} from EQE spectra (J_{cal}) for PM6:Qx-p-4Cl slot-die-coated semitransparent devices.

Samples	J_{cal} (mA cm^{-2})
1 cm^2 device with 1:1 ratio w/o ARC	20.31
1 cm^2 device with 1:2 ratio w/o ARC	20.10
1 cm^2 device with 1:3 ratio w/o ARC	19.04
1 cm^2 device with 1:4 ratio w/o ARC	17.24
1 cm^2 device with 1:5 ratio w/o ARC	15.33
1 cm^2 device (119 nm) with 1:3 ratio and 35 nm ARC	16.63
1 cm^2 device (180 nm) with 1:3 ratio and 35 nm ARC	18.18
1 cm^2 device (195 nm) with 1:3 ratio and 35 nm ARC	18.72
1 cm^2 device (206 nm) with 1:3 ratio and 35 nm ARC	18.87
1 cm^2 device (260 nm) with 1:3 ratio and 35 nm ARC	20.41
1 cm^2 device (301 nm) with 1:3 ratio and 35 nm ARC	20.51
0.0256 cm^2 device with 1:3 ratio and 35 nm ARC	19.00

Supplementary Table 8. The AVT values of spin-coated active layer (1:1.5) and slot-die-coated active layer (1:3) with different thicknesses.

Processing methods	Thickness (nm)	AVT (%)
Spin coating (D:A ratio = 1:1.5)	126	41.33
	93	50.25
	82	56.43
	67	58.32
	56	60.89
	41	65.47
	37	68.03
	33	71.04
Slot-die coating (D:A ratio = 1:3)	205	54.69
	170	61.22
	125	69.57
	106	72.12

		98	73.58

Supplementary Table 9. The GIWAXS parameters of π-π stacking of PM6:Qx-p-4Cl in BHJ films.

Processing Methods	D:A ratios	Position of π-π stacking ($Å^{-1}$)	d (Å)	FWHM ($Å^{-1}$)	CCL (Å)
Spin coating	1:0.5	1.71	3.67	0.25	25.25
	1:1.5	1.71	3.68	0.23	27.81
	1:3	1.69	3.71	0.21	30.41
Slot-die coating	1:1	1.72	3.66	0.26	24.17
	1:3	1.72	3.66	0.24	26.10
	1:5	1.71	3.67	0.22	28.06

Supplementary Table 10. The parameters from AFM for PM6:Qx-p-4Cl.

Processing methods	D:A ratios	R_q (nm)
Spin coating	1:0.5	3.29
	1:1.5	6.02
	1:3	8.57
Slot-die coating	1:1	3.17
	1:3	2.25
	1:5	1.75

Supplementary Table 11. The half-time of rising of hole transfer ($t_{half\text{-}time}$).

Processing methods	D:A ratios	$t_{half\text{-}time}$ (ps)
Spin coating	1:0.5	0.39
	1:1.5	0.50
	1:3	0.61
Slot-die coating	1:1	0.36
	1:3	0.32
	1:5	0.27

Supplementary Table 12. The fitted lifetimes of LE state.

Processing methods	D:A ratios	t1 (ps)	A1 (%)	t2 (ps)	A2 (%)	t3 (ps)	A3 (%)	t4 (ps)	A4 (%)
Spin coating	1:0.5	0.29	62.1	3.53	26.2	83.9	9.2	Inf	2.6
	1:1.5	0.42	52.9	4.25	32.0	68.1	12.7	Inf	2.4

	1:3	0.58	55.5	4.64	29.9	47.4	14.5	Inf	0.14
Slot-die coating	1:1	0.43	54.4	2.21	33.3	24.0	11.4	Inf	0.9
	1:3	0.30	49.2	2.19	36.7	55.2	10.9	Inf	3.2
	1:5	0.30	57.6	2.50	29.1	24.3	10.8	Inf	2.5

Supplementary Table 13. The fitted lifetimes of polaron state.

Processing methods	D:A ratios	t1 (ps)	A1 (%)	t2 (ps)	A2 (%)	t3 (ps)	A3 (%)	t4 (ps)	A4 (%)
Spin coating	1:0.5	4.62	36.1	76.8	26.6	1360	22.0	Inf	15.3
	1:1.5	3.44	45.0	39.8	29.0	1010	17.2	Inf	8.8
	1:3	2.11	50.7	22.3	39.0	745	9.8	Inf	0.5
Slot-die coating	1:1	2.74	31.1	36.4	38.9	788	15.8	Inf	14.2
	1:3	2.47	32.2	34.3	32.8	821	20.9	Inf	14.1
	1:5	2.52	35.5	34.5	40.7	631	18.8	Inf	5.0

Supplementary Table 14. Fitting parameters for Qx-p-4Cl, Y6, L8-BO and Qx-1 fluence measurements.

Acceptor	Parameter (excitation density)	Value ($\times 10^{18}$ cm^{-3})	γ ($\times 10^{-8}$ s-1)
Qx-p-4Cl	n_1	1.75	6.09
	n_2	4.09	7.33
	n_3	4.38	8.26
Y6	n_1	1.00	4.24
	n_2	1.06	4.66
	n_3	1.60	4.88
L8-BO	n_1	5.59	9.74
	n_2	5.81	10.34
	n_3	9.59	11.13
Qx-1	n_1	3.20	11.92
	n_2	4.55	12.96
	n_3	6.34	13.65

Supplementary Table 15. The Fitted L_D parameters.

Acceptor	L_D (nm)
Qx-p-4Cl	22.34
Y6	25.01

L8-BO	32.29
Qx-1	37.04

Supplementary Table 16. The fitted lifetime of PL decays of acceptor in corresponding blends.

Acceptor	D:A ratio	t_1 (ps)
Qx-p-4Cl	1:0.5	109.93
	1:1.5	112.44
	1:3	115.36
Y6	1:0.5	283.72
	1:1.5	294.62
	1:1.5	304.51
L8-BO	1:0.5	124.32
	1:1.5	147.63
	1:1.5	168.81
Qx-1	1:0.5	128.45
	1:1.5	188.15
	1:1.5	222.44

Supplementary Table 17. The detailed parameters of semitransparent modules based on donor-diluted PM6:Qx-p-4Cl system (D:A = 1:3) with effective area of 100 cm^2.

Area (cm^2)	V_{OC} (V)	J_{SC} (mA cm^{-2})	FF (%)	PCE (%)	AVT (%)	LUE (%)
100	18.85	0.79	69.85	10.40 (±0.25)	31.97	3.32

Supplementary Table 18. The summary of the viscosity of PM6:Qx-p-4Cl with different D:A ratios.

D:A ratios	Viscosity (mPa s)
1:0.5	5.95
1:1	3.55
1:1.5	2.91
1:3	1.85
1:5	1.44

Supplementary Table 19. The device parameters of ST-modules in reported works in Supplementary Figure 33.

Num.	Area (cm^2)	LUE (%)	Reference
1	10.80	2.02	[2]
2	10.17	1.10	[4]
3	12.80	1.67	[5]

4	12.80	3.05	
5	12.80	1.69	
6	19.30	2.60	6
7	21.00	3.30	7
8	100.00	2.20	
9	30.00	1.61	8
10	114.50	0.90	9
11	18.00	1.30	10
12	47.04	1.05	11
13	21.28	1.36	
14	10.30	1.62	12
15	10.30	0.89	
16	179.00	1.90	13

Supplementary Table 20. The CTM of PCE of opaque OSCs, which is calculated from equation of $CTM_{PCE} = PCE_{monolithic\ device}/PCE_{module}$, in reported works in Supplementary Figure 34.

Num.	Monolithic device		Module		CTM_{PCE} (%)	Ref.
	Area (cm^2)	PCE (%)	Area (cm^2)	PCE(%)		
1	1	17.55	11.08	16.10	92	14
2	0.05	17.88	15.40	15.94	89	15
3	1	16.20	41.00	14.04	87	16
4	1	11.32	108.00	9.15	81	17
5	0.06	19.00	19.30	16.26	86	18
6	1	14.25	46.20	13.25	93	19
7	1	14.82	28.00	13.25	89	20
8	1	14.82	52.00	13.26	90	
9	1	14.82	80.00	13.13	89	
10	1	14.82	104.00	13.00	88	

Supplementary Table 21. The CTM of PCE and LUE of ST-OSCs, which is calculated from the equation of $CTM_{PCE} = PCE_{monolithic\ device}/PCE_{module}$ and $CTM_{LUE} = LUE_{monolithic\ device}/LUE_{module}$, respectively.

Num.	Monolithic device			Module			CTM_{PCE} (%)	CTM_{LUE} (%)	Ref.
	Area (cm^2)	PCE (%)	LUE (%)	Area (cm^2)	PCE (%)	LUE (%)			
1	0.09	12.27	2.50	10.8	9.54	2.02	77.75	80.80	2
2	0.09	12.27	2.50	3.6	9.74	2.06	79.38	82.60	

3	0.04	8.30	1.90	12.8	7.5	1.67	90.36	87.89	[5]
4	1.4	7.9	4.05	7.14	5.25	2.63	66.46	64.94	[21]
5	-	12.6	3.9	21	10.2	3.3	80.95	84.62	[7]
6	-	12.6	3.9	100	6.7	2.2	53.17	56.41	
7	1.05	2.93	2.06	10.3	2.31	1.62	78.84	79.17	[12]
8	1.05	1.37	1.10	10.3	1.12	0.89	81.75	82.06	
9	0.063	13.31	6.02	16.8	11.3	4.53	84.90	75.25	[22]
10	-	12.78	2.68	19.3	11.28	2.26	88.26	84.02	[23]
11	0.04	12.07	5.04	120	6.69	2.70	55.43	53.57	[24]
12	0.0256	12.37	4.00	100	10.40	3.32	84.07	83.00	This work
13	1	11.81	3.90	100	10.40	3.32	88.06	85.13	

Supplementary Table 22. The summary of the temperature inside the house model at different irradiation time.

Irradiation time (min)	Temperature (℃)
0	21.3
3	21.6
6	21.8
9	22.3
12	22.9
15	23.5
18	24.1
21	24.9
24	25.5
27	25.9
30	26.2
33	26.5
36	28.1
39	29.1
42	30.5
45	31.6
48	32.6
51	33.3
54	34
57	34.6
60	35.2
63	35.7

Supplementary Table 23. The summary of weather data during the outdoor stability testing period.

Date	Weather	Temperature (°C)	Humidity (%)
2025/10/28-2025/11/3	Cloudy turning into clear skies	1-18	25-60
2025/11/4-2025/11/10	Light rain turning into clear skies	0-14	20-88
2025/11/11-2025/11/17	Clear skies	-4-17	17-39
2025/11/18-2025/11/24	Clear skies	-4-15	20-28
2025/11/25-2025/12/1	Clear skies	-6-9	17-37
2025/12/2-2025/12/9	Clear skies	-8-9	18-40

Supplementary References

1. Wang, Y. et al. Top and Bottom Electrode Optimization Enabled High-Performance Flexible and Semi-Transparent Organic Solar Cells. *Mater. Chem. Front.* **5**, 4310-4316 (2021).

2. Zha, W. et al. Bulk Contact Between Silver Nanowires Top Electrode and Interface Layer Enables High Performance of Full-Solution-Processed Semitransparent Organic Solar Cell Module. *Sol. RRL* **7**, 2300322 (2023).

3. Guan, S. et al. Balancing the selective absorption and photon-to-electron conversion for semitransparent organic photovoltaics with 5.0% light-utilization efficiency. *Adv. Mater.* **34**, e2205844 (2022).

4. Wachsmuth, J. et al. Fully Printed and Industrially Scalable Semitransparent Organic Photovoltaic Modules: Navigating through Material and Processing Constraints. *Sol. RRL* **7**, 2300602 (2023).

5. Huang, X., Fan, D., Li, Y. & Forrest, S. R. Multilevel peel-off patterning of a prototype semitransparent organic photovoltaic module. *Joule* **6**, 1581-1589 (2022).

6. Fan, J. Y. et al. High-performance organic solar modules via bilayer-merged-annealing assisted blade coating. *Adv. Mater.* **34**, 2110569 (2022).

7. Xie, J. et al. Multifunctional ternary semitransparent organic solar cell module with area above 100 cm^2 and average visible transmittance above 30%. *Energy Environ. Sci,* **17**, 7681-7690 (2024).

8. Han, Y. W., Lee, H. S. & Moon, D. K. Printable and semitransparent nonfullerene organic

solar modules over 30 cm(2) introducing an energy-level controllable hole transport layer. *ACS Appl. Mater. Interfaces* **13**, 19085-19098 (2021).

9. Berny, S. et al. Solar trees: first large-scale demonstration of fully solution coated, semitransparent,flexible organic photovoltaic modules. *Adv. Sci.* **3**, 1500342 (2016).

10. Dong, S. et al. Single-component non-halogen solvent-processed high-performance organic solar cell module with efficiency over 14%. *Joule* **4**, 2004-2016 (2020).

11. Tisao, C.-S. et al. Lab-to-Fab development and long-term greenhouse test of stable flexible semitransparent organic photovoltaic module. *Mater. Today Energy* **36**, 101340 (2023).

12. Meng, R., Jiang, Q. & Liu, D. Balancing efficiency and transparency in organic transparent photovoltaics. *npj Flex. Electron.* **6**, 39 (2022).

13. Jeong, H.-S. et al. Realizing the potential of scalable and stable semitransparent organic solar modules. *Chem. Eng. J.* **503**, 158497 (2025).

14. Feng, E. et al. A 16.10% efficiency organic solar module with ultra-narrow interconnections fabricated via nanosecond ultraviolet laser processing
Cell Rep. Phys. Sci. **5**, 101883 (2024).

15. Wang, S. et al. Designing dithieno-benzodithiophene-based small molecule donors for thickness-tolerant and large-scale polymer solar cells. *Energy Environ. Sci.* **17**, 2610-2620 (2024).

16. Lu, X. et al. Increase in the efficiency and stability of large-area flexible organic photovoltaic modules via improved electrical contact. *Nat. Energy* **9**, 793-802 (2024).

17. Kwon, H. C. et al. Overcoming the low-surface-energy-induced wettability problem of flexible and transparent electrodes for large-area organic photovoltaic modules over 500 cm2. *Adv. Energy Mater.* **12**, 2200023 (2022).

18. Chen, T. et al. Delayed crystallization kinetics allowing high-efficiency all-polymer photovoltaics with superior ipscaled manufacturing. *Adv. Mater.* **36**, 2308061 (2024).

19. Wang, C. et al. Unique assembly of giant star-shaped trimer enables non-halogen solvent-fabricated, thermal stable, and efficient organic solar cells. *Joule* **7**, 2386-2401 (2023).

20. Yang, S. et al. High cell to module efficiency remaining ratio of approximately 90% for the 100 cm(2) fully roll-to-roll gravure printed flexible organic solar cells from non-halogenated

solvent. *Adv. Mater.* 2500115 (2025).

21. Duan, X. et al. Solid additive dual-regulates spectral response enabling high-performance semitransparent organic solar cells. *Adv. Mater.* **36**, 2308750 (2024).

22. Ding, J. et al. Manipulating molecular stacking for semitransparent organic photovoltaics achieving light utilization efficiency >6. *Adv. Mater.* **37**, 2420439 (2025).

23. Wang, D. et al. High-performance see-through power windows. *Energy Environ. Sci.* **15**, 2629-2637 (2022).

24. Xie, D. et al. Scalable polymer for large-area semitransparent organic photovoltaics. *Joule* **9**, 102173 (2025).
