# Supplementary material for: Scalable semitransparent organic solar cells with robust film thickness tolerance for building-integrated photovoltaics
Source: Nat Commun. 2026 Feb 18;17:2916. doi: 10.1038/s41467-026-69537-3 (PMC13031838; doi:10.1038/s41467-026-69537-3)
Supplement: Supplementary file 2 — Description of Additional Supplementary Files [file 41467_2026_69537_MOESM2_ESM.pdf]

Description of Additional Supplementary Information

Title: Supplementary Movie 1

Description: Driving LCD screen via a 600 cm^2 power-generating window in this study.
