# Supplementary material for: Scalable semitransparent organic solar cells with robust film thickness tolerance for building-integrated photovoltaics
Source: Nat Commun. 2026 Feb 18;17:2916. doi: 10.1038/s41467-026-69537-3 (PMC13031838; doi:10.1038/s41467-026-69537-3)
Supplement: Supplementary file 4 — Reporting Summary [file 41467_2026_69537_MOESM4_ESM.pdf]

nature research

Corresponding author(s): Prof. Zhixiang Wei(weizx@nanoctr.cn)

Solar Cells Reporting Summary

Nature Research wishes to improve the reproducibility of the work that we publish. This form is intended for publication with all accepted papers reporting the characterization of photovoltaic devices and provides structure for consistency and transparency in reporting. Some list items might not apply to an individual manuscript, but all fields must be completed for clarity.

For further information on Nature Research policies, including our data availability policy, see Authors & Referees.

ü Experimental design

Please check: are the following details reported in the manuscript?

1. Dimensions

Item	Yes/No	Details
Area of the tested solar cells	[x] Yes [ ] No	0.0256 cm2 for small-area device; 1 cm2 for large-area device; 100 cm2 for large-area module
Method used to determine the device area	[x] Yes [ ] No	The masks with calibrated area were used to determine the device area.

2. Current-voltage characterization

Item	Yes/No	Details
Current density-voltage (J-V) plots in both forward and backward direction	[ ] Yes [x] No	Hysteresis has not been observed in the organic solar cells (OSCs).
Voltage scan conditions *For instance: scan direction, speed, dwell times*	[x] Yes [ ] No	The scanning step was 0.01 V and dwell time was 30 ms.
Test environment *For instance: characterization temperature, in air or in glove box*	[x] Yes [ ] No	The large-area devices were tested in air and the small-area devices were tested in a nitrogen atmosphere. The average temperature and relative humidity were 25°C and 30%, respectively.
Protocol for preconditioning of the device before its characterization	[ ] Yes [x] No	No preconditioning was applied prior to device characterization.
Stability of the J-V characteristic *Verified with time evolution of the maximum power point or with the photocurrent at maximum power point; see ref. 7 for details.*	[x] Yes [ ] No	The MPP tracking was undertaken for the photostability test.

3. Hysteresis or any other unusual behaviour

Item	Yes/No	Details
Description of the unusual behaviour observed during the characterization	[ ] Yes [x] No	No hysteresis observed during the characterization in the manuscript.
Related experimental data	[ ] Yes [x] No	No experimental data related.

4. Efficiency

Item	Yes/No	Details
External quantum efficiency (EQE) or incident photons to current efficiency (IPCE)	[x] Yes [ ] No	EQE spectra of all the devices were put in supplementary Figure 6-10.
A comparison between the integrated response under the standard reference spectrum and the response measure under the simulator	[x] Yes [ ] No	The integrated JSC calculated from the EQE curves are comparable to those obtained from the J-V curves.
For tandem solar cells, the bias illumination and bias voltage used for each subcell	[ ] Yes [x] No	No tandem cells were investigated in the manuscript.

5. Calibration

Item	Yes/No	Details
Light source and reference cell or sensor used for the characterization	[x] Yes [ ] No	The J–V measurement was tested via a Keithley 2400 sourcemeter. A solar simulator (SAN-EI ELECTRIC XES-1004SE-200S) with AM 1.5G spectra at 100 mW/cm². The light intensity was calibrated by the certified standard silicon solar cell (Newport Oriel 91150V-KG5).

Confirmation that the reference cell was calibrated and certified	[x] Yes [ ] No	The reference cell with KG5 filter was purchased from Newport Co., Ltd. and was calibrated and certified.
Calculation of spectral mismatch between the reference cell and the devices under test	[x] Yes [ ] No	Spectral mismatch between reference cell and testing cells was less than 5%.

6. Mask/aperture

Size of the mask/aperture used during testing	[x] Yes [ ] No	Size of mask used during the testing is 0.0256 cm2 and 1 cm2 for small-area and large-area devices, respectively.
Variation of the measured short-circuit current density with the mask/aperture area	[ ] Yes [x] No	Since this work is mainly focused on structural characterization and discussion of the applications of BIPVs, we did not test our fabricated cells under various mask area.

7. Performance certification

Identity of the independent certification laboratory that confirmed the photovoltaic performance	[ ] Yes [x] No	Since this work is mainly focused on structural characterization and discussion of the applications of BIPVs, we did not certificate our measured photovoltaic performance.
A copy of any certificate(s)<br>*Provide in Supplementary Information*	[ ] Yes [x] No	Since this work is mainly focused on structural characterization and discussion of the applications of BIPVs, we did not certificate our measured photovoltaic performance.

8. Statistics

Number of solar cells tested	[x] Yes [ ] No	At least 5 independent solar cells fabricated under the same conditions were tested.
Statistical analysis of the device performance	[x] Yes [ ] No	Statistical results of device performance can be found in supplementary table 2-6.

9. Long-term stability analysis

Type of analysis, bias conditions and environmental conditions<br>*For instance: illumination type, temperature, atmosphere humidity, encapsulation method, preconditioning temperature*	[x] Yes [ ] No	For the photostability test of 1 cm2 ST-device, the ST-device was measured under 100 mW cm-2 illumination from an LED light source in nitrogen atmosphere, and the temperature was maintained at 20℃.<br>For outdoor stability test of 100 cm2 ST-module, the module is encapsulated using UV-curable adhesive and glass, and the detailed weather information were listed in Supplementary Table 23.
